# The Probability to Initiate X Chromosome Inactivation Is Determined by the X to Autosomal Ratio and X Chromosome Specific Allelic Properties

**DOI:** 10.1371/journal.pone.0005616

**Published:** 2009-05-19

**Authors:** Kim Monkhorst, Bas de Hoon, Iris Jonkers, Eskeatnaf Mulugeta Achame, Wouter Monkhorst, Jos Hoogerbrugge, Eveline Rentmeester, Hans V. Westerhoff, Frank Grosveld, J. Anton Grootegoed, Joost Gribnau

**Affiliations:** 1 Department of Reproduction and Development, Erasmus MC, University Medical Center, Rotterdam, the Netherlands; 2 Department of Cell Biology, Erasmus MC, University Medical Center, Rotterdam, the Netherlands; 3 Code-B Internet Applications, Delft, the Netherlands; 4 Department of Molecular Cell Physiology, Free University Amsterdam, Amsterdam, the Netherlands; 5 Manchester Centre for Integrative Systems Biology, Manchester, United Kingdom; Oregon State University, United States of America

## Abstract

**Background:**

In female mammalian cells, random X chromosome inactivation (XCI) equalizes the dosage of X-encoded gene products to that in male cells. XCI is a stochastic process, in which each X chromosome has a probability to be inactivated. To obtain more insight in the factors setting up this probability, we studied the role of the X to autosome (X∶A) ratio in initiation of XCI, and have used the experimental data in a computer simulation model to study the cellular population dynamics of XCI.

**Methodology/Principal Findings:**

To obtain more insight in the role of the X∶A ratio in initiation of XCI, we generated triploid mouse ES cells by fusion of haploid round spermatids with diploid female and male ES cells. These fusion experiments resulted in only XXY triploid ES cells. XYY and XXX ES lines were absent, suggesting cell death related either to insufficient X-chromosomal gene dosage (XYY) or to inheritance of an epigenetically modified X chromosome (XXX). Analysis of active (Xa) and inactive (Xi) X chromosomes in the obtained triploid XXY lines indicated that the initiation frequency of XCI is low, resulting in a mixed population of XaXiY and XaXaY cells, in which the XaXiY cells have a small proliferative advantage. This result, and findings on XCI in diploid and tetraploid ES cell lines with different X∶A ratios, provides evidence that the X∶A ratio determines the probability for a given X chromosome to be inactivated. Furthermore, we found that the kinetics of the XCI process can be simulated using a probability for an X chromosome to be inactivated that is proportional to the X∶A ratio. These simulation studies re-emphasize our hypothesis that the probability is a function of the concentration of an X-encoded activator of XCI, and of X chromosome specific allelic properties determining the threshold for this activator.

**Conclusions:**

The present findings reveal that the probability for an X chromosome to be inactivated is proportional to the X∶A ratio. This finding supports the presence of an X-encoded activator of the XCI process.

## Introduction

In placental mammals, dosage compensation of X-encoded gene products is achieved by inactivation of either of the two X chromosomes in female cells [1]. Random X chromosome inactivation (XCI) is initiated early during female embryonic development, and results in a transcriptionally inactive X chromosome (Xi). The inactive state of the Xi is clonally propagated through many cell divisions. At the onset of XCI the X-linked non-coding *Xist* gene is transcriptionally up-regulated on the future Xi, and *Xist* RNA coats the Xi in *cis* [2]–[5]. *Xist* RNA is required for XCI and most likely attracts chromatin modifying enzymes involved in the silencing process [6], [7]. The *Tsix* and *Xite* genes play a crucial role in the early stages of XCI by suppression of *Xist* transcription and *Xist* RNA accumulation. Both *Tsix* and *Xite* also are non-coding genes that overlap with *Xist*, but are transcribed in anti-sense direction [8], [9].

The first phase of XCI comprises a counting process, followed by initiation of XCI when more than one X chromosome is present per diploid nucleus. We have recently shown that initiation of XCI is directed by a stochastic mechanism, in which all X chromosomes in a nucleus have an independent probability to initiate XCI within a certain time-span [10]. We proposed that this probability is proportional to the X to autosome ratio (X∶A), and most likely depends on at least two factors that act through *Xist* and *Tsix*: an X-encoded XCI-activator that stimulates *Xist* expression, and itself is transcriptionally inactivated by the XCI process, and an autosomally encoded XCI-inhibitor that suppresses *Xist* by activating *Tsix*. Although the action of *Tsix* is still not understood, *Tsix* transcription and chromatin modifications in the *Xist* promoter (possibly mediated by *Tsix*) provide a threshold that has to be overcome by the XCI-activator, allowing accumulation of sufficient *Xist* molecules to silence *Tsix* and spread in *cis*. Early in mouse development or upon differentiation of embryonic stem (ES) cells, the XCI-activator concentration in a cell will increase, and in female cells this will drive the initiation of XCI with a specific probability. This probability is the consequence of stochastic transcriptional activation of both *Xist* and *Tsix*. In male cells, the XCI-activator concentration will be too low; therefore, these cells induce XCI only sporadically [10].

Several findings support the presence of an X-linked XCI-activator. Tetraploid XXXX ES cells initiate XCI significantly faster than XXXY cells [10]. In addition, female ES cells with a heterozygous deletion including *Xist*, *Tsix* and *Xite* (ΔXTX), still show initiation of XCI on the wild type X chromosome. XCI is not initiated in male cells with one copy of *Xist*, *Tsix* and *Xite* indicating a novel *trans* acting activator, encoded by a gene located outside the deleted area [10]. Also, studies in differentiating ES cell lines with stably integrated *Xist* promoter transgenes show significantly more expression of a linked reporter in female cells compared to male cells [11]. The genomic location of the XCI-activator is unknown sofar. However, previous studies which analyzed XCI in male cell lines with multi-copy YAC transgenes ranging in size from 320 to 460 kb, encompassing *Xist* and flanking regions, revealed initiation of XCI on the single X chromosome [12], [13]. Interestingly, a BAC sequence covering a region upstream of *Xist*, not including *Xist* itself, also induced ectopic XCI in transgenic male and female cells [14]. These studies indicate that the sequence encoding the XCI-activator is likely to be located within the sequence covered by these transgenes. Smaller transgenes, only including *Xist* and flanking sequences, have also been reported to induce ectopic XCI in male cells, when present as multiple tandemly inserted transgenes [15], [16]. Our finding that XCI is still initiated in female cells with a ΔXTX deletion, however, indicates that the overlapping region covered by the ΔXTX deletion and these transgenes [15], [16] is not required for the counting process. Some of the reported observations may also be attributed to the presence of *Tsix* transcription, which was not yet discovered, and hence not taken into consideration, at the time these studies were performed.

In diploid and tetraploid cells, one X chromosome will remain active per diploid genome. However, in triploid cells this ratio of one active X chromosome per diploid autosomal set cannot be achieved. Therefore, triploid cells provide a unique situation for studying the mechanism of XCI counting and choice and gene dosage related cell selection. Several studies have been conducted with human and mouse XXY and XXX triploid embryos and embryo-derived cell lines, to try to determine the pattern of X inactivation. In these experiments cultured differentiated cells were examined which had completed the XCI process [17], and indicated that the majority of cell lines derived from human live born XXX triploids predominantly show two active X chromosomes [18]–[20]. In contrast, analysis of 10-day-old XXY and XXX mouse triploid embryos showed that most cells had one active X chromosome [21]. Unfortunately, both studies did not discriminate between primary choice in XCI and the effect of cell selection processes on XCI.

To explore the mechanism determining the probability of an X chromosome to be inactivated, we have generated XXY triploid mouse ES cells. Analysis of XCI in these cells allowed us to determine the influence of the X∶A ratio on the initiation of XCI, and to discriminate between the effects of XCI initiation and cell selection. In addition, we have used stochastic and mathematical simulation studies to follow the kinetics of XCI in a population of developing or differentiating cells.

## Results

### Generation of triploid ES cells

Our previous studies with tetraploid XXXX, XXXY and XXYY mouse ES cell lines have indicated that the probability for an X chromosome to be inactivated is directly related to the X∶A ratio [10]. To further explore this finding we aimed to generate triploid ES cell lines with XYY and XXY karyotypes, having an X∶A ratio of 1∶3 and 2∶3, respectively, for which XCI has not been studied before. To generate triploid ES cell lines we decided to fuse puromycin resistant female and male ES cells with round spermatids or spermatozoa containing a *neomycin resistance* (*neo*) gene targeted to either the autosomal *Ube2b* gene or the X-chromosomal *Ube2a* gene. Both *Ube2a* and *Ube2b* encode ubiquitin-conjugating enzymes involved in DNA replicative damage bypass [22]. The encoded proteins have at least partially overlapping functions, and two functional alleles of either *Ube2a* or *Ube2b* per cell are sufficient to generate viable diploid mice. Also, spermatogenesis is not dysregulated in *Ube2a* knockout and *Ube2b* heterozygous mutant mice [23], [24]. Therefore, it was expected that loss-of-function of one targeted *Ube2a* or *Ube2b* allele, in the *Ube2b* ^+/−^ and *Ube2a* ^y/−^ mice, respectively, will not have an effect on the viability of hybrid fusion products. In the present study, the targeted mutant alleles serve the function of selection for fused cells.

**Figure 1:**
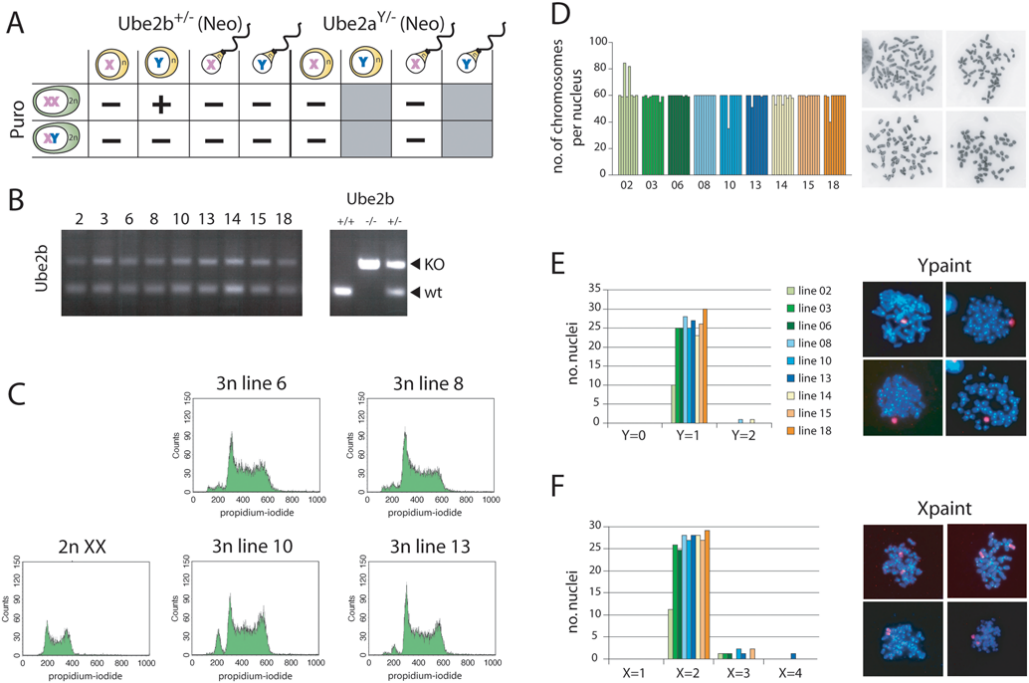
Generation of triploid ES cells. A) The different fusion experiments performed; (−) no clones present, (+) clones present which could be picked and expanded. Fusion of Y-bearing spermatids and spermatozoa for *Ube2a* knockout mice was not examined, since the neo selection marker localizes to the X (gray boxes). B) PCR with genomic DNA detecting the wild type and mutated *Ube2b-neo* allele. Clone numbers are indicated, and control DNA was isolated from wild type, *Ube2b*−/− and +/− mice. C) FACS analysis detecting the DNA content of diploid ES cells, and four different triploid ES cell lines analyzed in (B). D) Karyotyping of 9 triploid ES cell lines, shown in (B); indicated are chromosome counts of individual methaphase spreads. Right panels show representative examples of metaphase spreads. E) Y chromosome paint analysis; shown is the number of metaphase spreads with 0, 1, and 2 Y chromosomes. Right panels show representative examples of metaphase spreads subjected to DNA FISH using a Y paint probe (red, DNA is blue). F) X chromosome paint analysis, shown is the number of metaphase spreads with 1, 2, 3, and 4 X chromosomes. Right panels show representative examples of metaphase spreads subjected to DNA FISH using an X paint probe (Red, DNA is blue).

All PEG mediated fusion experiments were conducted twice. Fusion of the neomycin resistant *Ube2a-neo* round spermatids and spermatozoa with female or male ES cells did not result in double resistant colonies (Figure 1A). In addition, fusions of *Ube2b-neo* round spermatids with male ES cells, and fusions of *Ube2b-neo* spermatozoa with either female or male ES cells, also did not result in double resistant colonies. In contrast, double resistant colonies were obtained by fusion of *Ube2b-neo* round spermatids with female ES cells, and these colonies were picked and expanded for further analysis. PCR analysis of genomic DNA indicated the presence of the *Ube2b-neo* allele in all the ES clones picked, confirming the fusion of a round spermatid containing the *Ube2b-neo allele* (Figure 1B). FACS analysis, using propidium iodide to determine the DNA content, indicated that all our cell lines were triploid (Figure 1C). The small 2n population we attribute to contamination of the triploid cells with diploid male feeders that we used to grow the ES cells on. Karyotyping also indicated that, in all cell lines, the majority of cells had 60 chromosomes, which are stably maintained through many passages (Figure 1D, and data not shown). Interestingly, X and Y chromosome paint analysis showed that all cell lines had an XXY 3n karyotype (N = 18) although the haploid round spermatids that were used for fusion can be expected to contain either an X chromosome or a Y chromosome in a 50/50 ratio (Figure 1E and 1F).

These results suggest that triploid XYY cells are absent due to an insufficient dosage of X-encoded genes. In addition, the lack of XXX 3n karyotypes suggests that introduction of an X chromosome through a round spermatid leads to a non-viable triploid ES cell. The absence of triploid XXX ES cell lines (X∶A ratio of 1) can not be explained by a dosage problem, but might be due to the presence of an epigenetically modified X chromosome present in spermatids. During spermatogenesis, the largely unpaired X and Y chromosomes are transcriptionally inactivated, forming the XY body or sex body in a process called meiotic sex chromosome inactivation (MSCI) [25]. Chromatin modifications present on the XY body may be partly maintained in post-meiotic round spermatids. Such modifications, particularly relevant for the X chromosome with a large gene content, may explain lethality of the triploid XXX ES cells.

To test whether the spermatid derived X was reactivated after cell fusion, we fused round spermatids of males containing an X-linked *GFP* transgene [26] with male and female ES cells. Analysis of diploid ES cells containing the X-linked *GFP* transgene shows robust GFP expression, indicating that the transgene is properly expressed in ES cells. In contrast, after fusion of *GFP* round spermatids with ES cells, we did not see reactivation of the transgene (data not shown). Selection for reactivation of the transgene by applying puromycin selection, at days 0, 3 and 5 after fusion, did not result in clones resistant to both selection reagents. Therefore, we conclude that ES cells are incapable of reactivating the X from spermatids. This is in contrast to ES fusions with female somatic cells, which lead to reactivation of the Xi [27].

### X chromosome inactivation in triploid XXY ES cells

**Figure 2:**
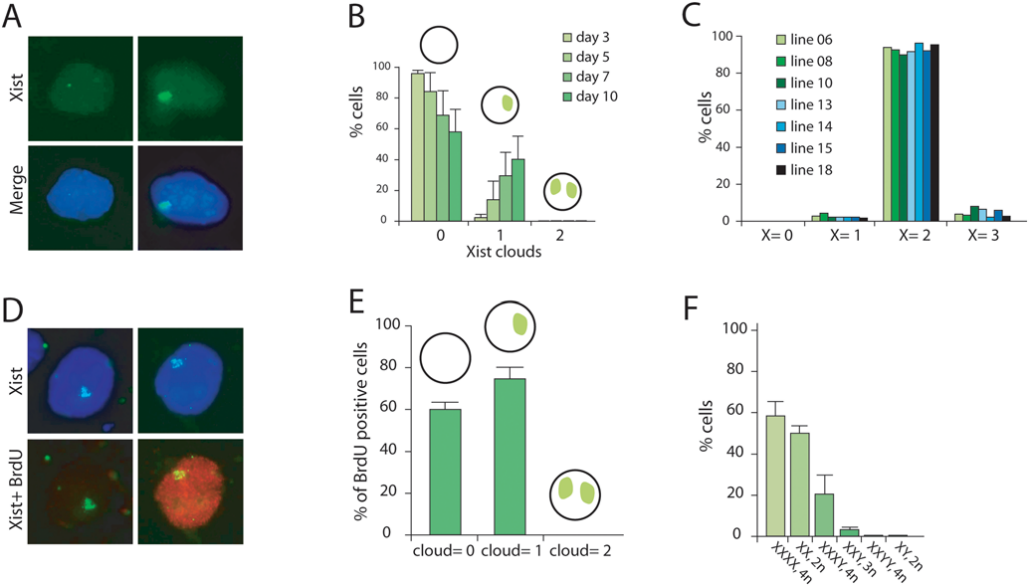
Analysis of XCI in differentiating triploid XXY ES cells. A) RNA FISH analysis with an *Xist* probe (FITC, DNA in blue) on day 3 differentiated triploid ES cells, shows cells with no (left panels) or one (right panels) *Xist* cloud. B) The average distribution and standard deviation of cells with different numbers of *Xist* clouds throughout differentiation. C) DNA FISH analysis on day 7 differentiated triploid ES cells. Shown is the relative number of cells with 0, 1, 2 and 3 X chromosomes. D) Combined *Xist* RNA-BrdU detection (*Xist* in FITC, BrdU in Rhodamine red, DNA in DAPI blue), indicating the presence of cells with negative and positive BrdU staining (from left to right). E) Quantification of the *Xist* RNA-BrdU detection, shown are the relative number and standard deviation of BrdU positive cells with 0, 1 and 2 *Xist* clouds. F) The relative number and standard deviation of cells that have initiated XCI (at least one Xi), per cell line at day 3 of differentiation.

To study XCI in the obtained XXY triploid ES cells, we differentiated 9 of these cell lines into embryoid bodies (EB). Cells were fixed and subjected to RNA-FISH after 3, 5, 7, and 10 days of differentiation, using an *Xist* specific probe to stain the *Xist* coated Xi's. After a three-day differentiation period, we mainly found cells with zero or one Xi, indicating that the triploid XXY cells are capable to initiate XCI (Figure 2A). At this time point only ∼2.5% of cells had one Xi (XaXiY) and we sporadically (<0.1%) found cells with two Xi's (XiXiY). During the differentiation process, the relative number of cells with one Xi increased, to ∼40% at day 10 of differentiation (Figure 2B).

To exclude the possibility that our triploid XXY ES cells lost or gained X chromosomes over the differentiation process, we performed DNA-FISH with an X chromosome specific BAC probe on cells differentiated for seven days. To obtain a reliable measurement, at least 100 nuclei were scored for every cell line. We found that over 93% of the XXY cells still contained two X chromosomes, and only 3% of the cells were found to have three X chromosomes, suggesting that the karyotype of these triploid XXY ES cells is stable throughout the differentiation period that we assayed (Figure 2C).

We further examined whether the increase in time of the percentage of XiXaY cells (Figure 2B) might be caused by cell selection. We therefore added BrdU 24 hours prior to cell fixation of day 7 differentiated ES cells, and performed immuno/RNA FISH, detecting BrdU positive cells and *Xist* RNA. Comparison of BrdU positive cells with one or no *Xist* cloud(s) shows that there are significantly more cells with one cloud, indicating that XiXaY cells indeed have a small but significant proliferative advantage (p<0.001; Figure 2D and 2E).

Previously, we have proposed that the probability for an X chromosome to be inactivated is proportional to the X∶A ratio [10]. To further explore this finding we compared the percentage of cells that had initiated XCI at day 3 of differentiation between cell lines with various X∶A ratios, notably our XXY triploid ES cell lines (with an X∶A ratio of 0.67) and tetraploid and diploid cells (4n XXXX cells with X∶A = 1; 4n XXXY cells with X∶A = 0.75; 4n XXYY cells with X∶A = 0.5; 2n XX cells with X∶A = 1; and 2n XY cells with X∶A = 0.5). ES cell lines were differentiated through EB differentiation and subjected to RNA FISH to detect *Xist* RNA. For each line with a different X∶A ratio or a different ploidy number, we performed three independent differentiation experiments.

The results (Figure 2F) confirm our previous findings [10] that, at day 3 of differentiation, XXXX cells have initiated XCI in significantly more cells (58%) than XXXY cells have (20%). Furthermore, tetraploid XXYY and diploid XY cells initiated XCI in less than 0.3% of the cells, whereas diploid XX ES cells initiated XCI in 50% of the cells (Figure 2F). At day 3, triploid XXY cells had initiated XCI in 3–4% of the cells (Figure 2F). This percentage falls between that found for XXXY and XXYY cells. From these results we conclude that the probability to initiate XCI depends on the X∶A ratio, and that this relationship appears not to be linear (Figure 2F).

### Parameters required for computer simulated XCI

To better understand the kinetics of XCI in a developing female embryo or a differentiating population of female ES cells we decided to simulate the XCI process. There are four important parameters required to simulate XCI, based on a stochastic model for XCI: 1) the probability for an X to initiate XCI, 2) the time window required for one choice round, 3) the rate of cell division, and 4) cell selection.

As indicated by our findings, the probability for an X to initiate XCI is proportional to the X∶A ratio, and XCI is most likely triggered by a threshold nuclear concentration of an X-encoded XCI-activator. Although the nuclear concentration of XCI-activator will be the same for both X chromosomes present in a female cell, specific allelic properties of the individual X chromosomes can result in different probabilities because of effects *in cis*. Previous studies with female ES cell lines harboring deletions of either *Xist*, *Tsix* or *Xite* have indicated that the probability to initiate XCI positively correlates with the *Xist* transcription rate and negatively with the transcription rate of *Tsix* and *Xite* [6]–[9], [28]. Based on their anti-sense nature, transcription initiated by the *Tsix* and *Xite* promoters may constitute a threshold for *Xist* to accumulate in *cis*. Also, *Xist* promoter related modifications could restrict the action of the XCI-activator and may therefore be involved in setting up the threshold. Only when sufficient XCI-activator is present in the nucleus enough *Xist* transcription is initiated to overcome *Tsix* mediated repression, thereby effectuating a probability to initiate XCI. *Xist* transcription initiation is a stochastic process itself, and depending on the nuclear XCI-activator concentration, bursts of *Xist* transcription will generate a continuum of small probabilities to initiate XCI in time. This probability will drop after inactivation of an X chromosome with the decline in the XCI-activator concentration that depends on the nuclear half life of the XCI-activator. In our simulations we have used a time window with a specified probability, which represents the integrated probability within that time window.

**Figure 3:**
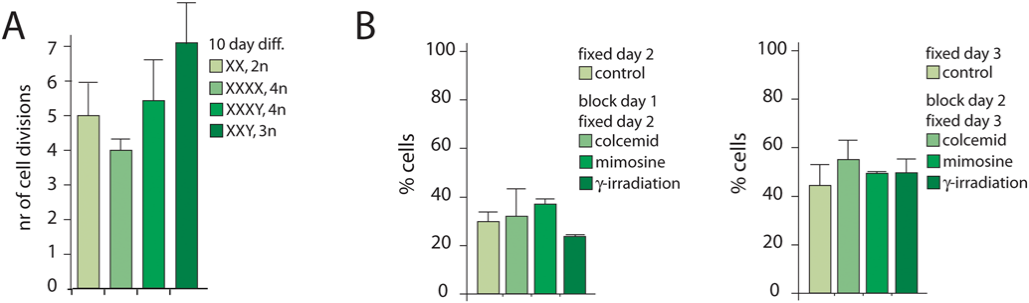
Initiation of XCI independent of cell division. A) Determination of the number of cell divisions during a 10 day EB differentiation period. Shown are the average and standard deviation of two separate experiments with two different ES lines per X∶A ratio or ploidy number. B) Analysis of initiation of XCI after a cell cycle block. Diploid female ES cells were EB differentiated for one day (left graph) or two days (right graph), and treated with colcemid or mimosine, or cells were lethally γ-irradiated, and allowed to differentiate for one more day before fixation. Control samples were allowed to differentiate for two or three days. The percentage of cells that initiated XCI was determined by *Xist* RNA FISH, followed by the quantification of the number of cells with *Xist* clouds.

To test whether XCI is dependent on cell division, we first analyzed the number of cell divisions during a 10 day period of embryoid body (EB) differentiation, for ES cells with different X∶A ratios. To determine the increase in cell number we differentiated 10^5^ cells, and isolated DNA before and after differentiation. OD measurements of two independent differentiation experiments indicated that the different ES lines divided between 4 and 7 times in the 10 day differentiation period (Figure 3A). Next, female diploid ES cells were EB differentiated for one or two days, and then subjected to γ-irradiation-, mimosine- or colcemid-mediated cell cycle arrests for one day. Initiation of XCI in treated and untreated cells was compared by counting the number of cells with or without an Xi, using RNA FISH with an *Xist* probe. We found no significant increase or decrease in the number of cells with an Xi after cell cycle arrest, although the cells that had been γ-irradiated at day 1 showed a slight decrease in cells that initiated XCI (Figure 3B). This result suggests that cell division is not required, or perhaps plays a minor role in the initiation of XCI. Nevertheless, cell division characteristics are important in the XCI process, because previous studies have shown that cells that inactivate too many X chromosomes stop dividing or slow down the cell division rate, which allows the cycling population of cells to outgrow the cells that inactivated too many X chromosomes [10], [29].

Cell selection also plays an important role in the XCI process, as male cells that inactivate their single X chromosome will die. Studies with inducible *Xist* cDNA transgenes integrated on the single X chromosome in male cells showed that *Xist* mediated silencing manifests within 24 hours, and that cell death becomes imminent within three days of *Xist* induction [29]. Also, diploid female cells inactivating two X chromosomes or incapable of initiating XCI are prone to die [6], [30].

### Computer simulated XCI

**Figure 4:**
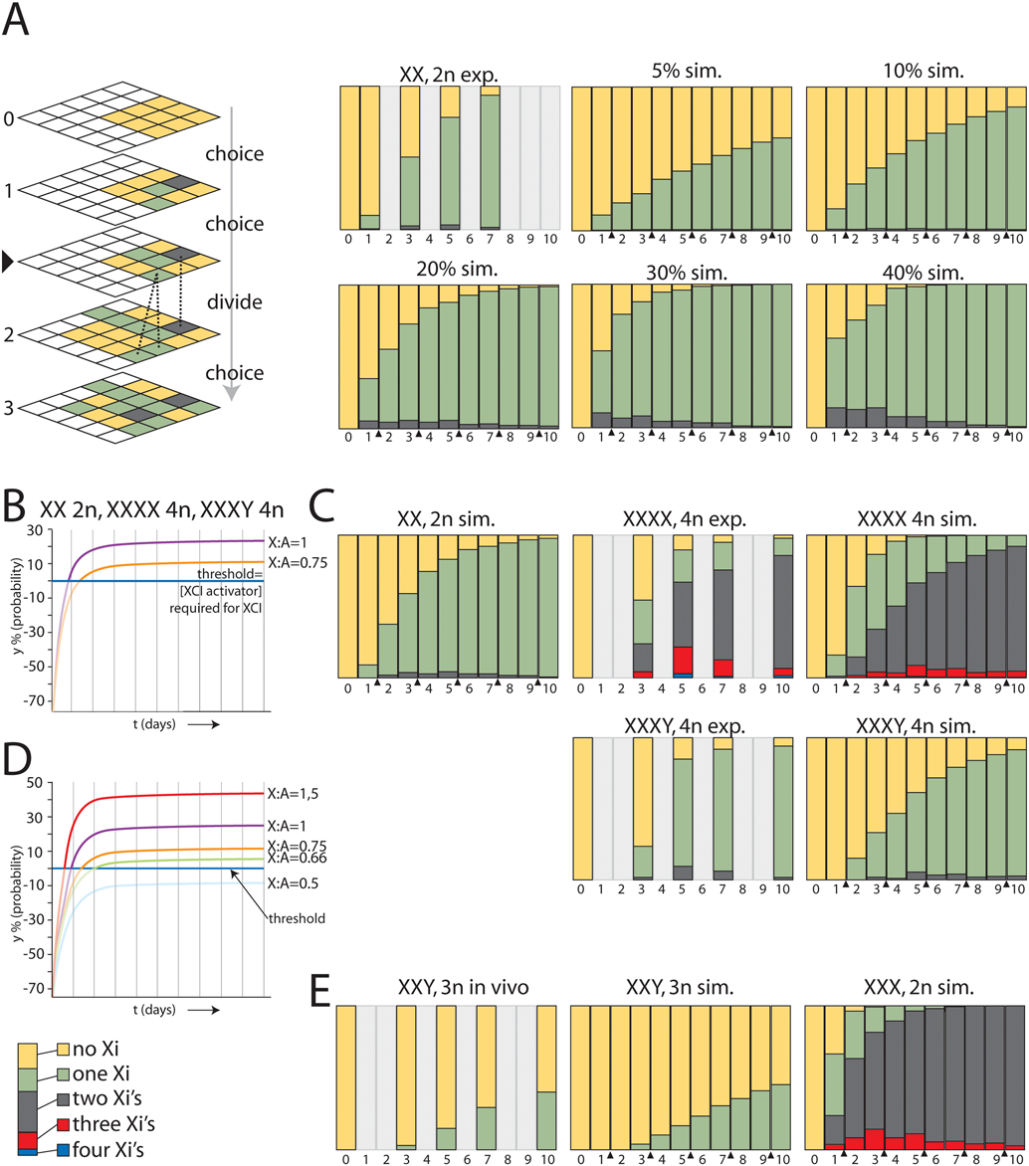
Computer simulation of XCI. A) Left upper panel shows a schematic representation of the stochastic simulation which is executed in a three dimensional matrix. Cells (boxes) go through consecutive choice rounds (numbers) interrupted by cell divisions (triangle). The three different cell types are represented by XaXa = yellow, XiXa = green, XiXi = gray boxes. The dashed lines indicate the origin of progeny of one XiXa and one XiXi cell after cell division (not all lines are shown, and note that the XiXi cells do not divide). The right panels show the experimental data from differentiated 2n female XX ES cells (exp.), and the stochastic simulation of XCI with a 5%, 10%, 20%, 30%, and 40% fixed probability per X chromosome (sim.). The different bar-graphs show the relative distribution of the three different cell types (XaXa = yellow, XiXa = green, XiXi = gray). Numbers below the bar graphs indicate days of differentiation (1–10), and cell division is indicated with a triangle. For time points represented by light gray bars no data is available. B) Probability curves representing the increase of the probability y in time based on equation [6], with m = 1, for XX 2n and XXXX 4n cells (purple), and m = 0,75 for XXXY 4n cells (orange). The probability at a given time point is the integrated probability over a time frame of one day. A negative value for y results in a probability of 0, and is represented by a faint line. C) Upper left panel shows simulation of XCI in XX diploid cells based on probabilities determined using different probabilities in time indicated in the curve, shown in (B). Upper middle panel shows the experimental percentages of 4n XXXX cells with a different number of Xi's throughout EB differentiation. The upper right panel shows the simulation of XCI using the same parameters as used for the XX diploid simulation (XaXaXaXa = yellow, XaXaXaXi = green, XaXaXiXi = grey, XaXiXiXi = red, XiXiXiXi, blue). Bottom left panel shows the experimentally determined percentages of 4n XXXY cells with a different number of Xi's throughout EB differentiation. Bottom right panel shows the XCI simulation of 4n XXXY cells using the different probabilities in time indicated in the curve presented in (B) (XaXaXaY = yellow, XaXaXiY = green, XaXiXiY = grey, XiXiXiY = red). D) Curves representing the probability y in time using equation [6] for cells with a different X∶A ratio, ranging from 0,5 to 1,5. E) Left panel shows the experimentally determined percentages of 3n XXY cells with a different number of Xi's throughout EB differentiation. Middle panel shows the XCI simulation of 3n XXY cells using different probabilities indicated in the curve presented in (D) (XaXaY = yellow, XaXiY = green, XiXiY = grey). Right panel shows the simulation of XCI in XXX 2n cells (XaXaXa = yellow, XaXaXi = green, XiXiXa = grey, XiXiXi, red).

To comprehend the kinetics of XCI, we have developed a stochastic simulation model to determine the populations of cells with a different number of Xa's and Xi's. In this approach, the distribution of different cell populations is derived using a simulation program with a random number generator, thereby mimicking the choice process. With a small starting population of cells, as present in the female mouse embryo around the time XCI is induced, the stochasticity resulted in different outcomes of the choice process for every new calculation. This phenomenon deviates from what would be obtained in deterministic mathematical models, but corresponds to experimental reality. Our stochastic simulation of the XCI process used a three-dimensional matrix in which each Z stack represented one choice round (Figure 4A). For our simulations we used a fixed or changing probability per choice round per X chromosome, and specific cell cycle characteristics depending on the X∶A ratio.

We started by simulating the XCI process in XX diploid female ES cells throughout a 10 day differentiation period, using a fixed probability in time to run the simulations, and compared the resulting distributions with experimental data obtained with differentiating XX female ES cells. For cells with an X∶A ratio≥0.5 we used a cell division rate of once every two days, based on our cell division analysis (described above). In addition, the simulation assumed that cells with all X chromosomes inactivated stop dividing, which is based on previous studies [31]. To be able to compare the simulations with available experimental data, we set the time window to 1 day, representing the integrated probability for cells choosing the X to be inactivated over 1 day. In the calculations we have excluded the option that, in XaXi cells that just have inactivated an X chromosome, the active second X chromosome may still have a probability to be inactivated. We performed the simulations with 100 cells, which mimics the number of cells present in the female mouse embryo around the time XCI is initiated. A number of 5 independent stochastic simulations generated the data for statistical analysis of the average and standard deviation. The graphs in the figures only show the average value for each time point. Comparison of the simulations, using an increasing range of fixed probabilities from 5% to 40% for both X-chromosomes, with previously obtained experimental results for differentiated XX female ES cells indicated that a fixed probability between 10% and 20% fits our experimental data best (Figure 4A; Figure S2A, B and C and Figure S3A, B and C) [10]. To validate the simulation results we also compared these with data obtained using a mathematical approach were fixed probabilities were used to calculate the different XaXa, XaXi and XiXi populations, which showed similar population dynamics (Text S1 and Figure S1). The fluctuation in the percentage of XiXi cells in time is the consequence of applying two choice rounds (1 per day) within one cell division (once every two days for XaXa and XaXi cells). Because cell division is synchronized in the simulations, XiXi cells are diluted out after every cell division.

From previous experimental data it appears that the probability to initiate XCI is lower in the beginning than later during the XCI process, and our experimental findings indicate that the probability is dependent on the concentration of XCI-activator in the nucleus. In our model, the labile XCI-activator is produced at a rate (v_a.synthesis_) that is proportional to the number of active X chromosomes. This leads to the following differential equation for the concentration of the XCI-activator:
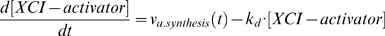
(1)with:
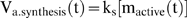
(2)Here, k_s_ is the rate constant for synthesis (in µMolar per second per active promoter), and m_active_ is the number of active X chromosomes per haploid genome, which can be substituted by the X∶A ratio by renormalizing the rate constant k_s_. Before XCI, i.e. at the start of the simulation, all X chromosomes are active, and whenever the number of active X chromosomes remains (approximately) constant in time, the above equation integrates as:
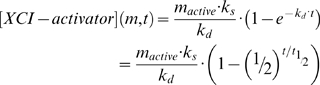
(3)where:
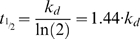
(4)We expect that the decision probability for X inactivation in any very short time period, is proportional to the concentration [y] of a number of molecules (or chromosome modifications) y that effectuate *Xist* promoter activation. These molecules are being synthesized through an enzymatic activity that depends on the concentration of the allosteric XCI-activator through a Hill relationship, with V_ys_ and K_a_, representing rate at maximum activator concentration and activation constants, respectively. Y is degraded by another enzymatic process, depending on the concentration y through a Michaelis Menten relationship, with V_yd_ and K_My_ and maximum rate and Michaelis constants, respectively. We anticipate that K_My_ is very small, whereby this degradation process of Y effectively always has the same rate. Also we expect that y has a certain constant lability, through a first order process, with first order rate constant k_dy_:
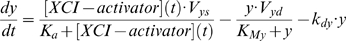
(5)These molecular processes will reach a quasi steady state. Equating dy/dt to zero, and assuming that K_My_ is much smaller than any relevant concentration of y, the concentration of y is given by:
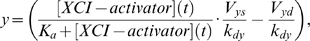
(6)or y will be zero if this yields a negative number. At the threshold level, y will be zero, resulting in:
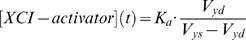
(7)Above the threshold level its value will increase with an increase in [XCI-activator] towards a maximum. By plotting y in time we generated a probability curve, which in the simulations represents the integrated probability over a time frame of 1 day. The values for y at different days from 0–10 were imported in the simulation program to assign the different Xi's with a specific probability per choice round. After XCI has started on one or more X chromosomes, the concentration of the XCI-activator will drop fairly quickly, according to the half-life of the XCI-activator protein (Text S1). For cells that started XCI in one choice round, the probability will drop according to the m value reached after that choice round.

Using graded preset values for the parematers, the outcome of simulations using the above model, was compared with experimental data sets that were obtained by differentiation of diploid XX (m = 1) and tetraploid XXXX (m = 1) and XXXY (m = 0.75) cells [10]. This approach enabled us to obtain best-fit values for the parameters (independent variables). Probability curves for m = 1 (2n XX and 4n XXXX) and m = 0.75 (4n XXXY) were derived (Figure S4A, B and C, and Figure 4B) with a K_a_ of 3.3 µM, equal to the maximum XCI-activator concentration in diploid XX and tetraploid XXXX cells, and values for k_s_, k_d_, k_dy_, V_ys_ and V_yd_ of 2 µM, 0.6 µM, 1.5 µM, 3 µM and 1.15 µM respectively. These curves resulted in simulated populations with different Xa's and Xi's in time that matched our experimental data with diploid XX and tetraploid XXXX, XXXY cells (Figure 4C, and Figure S5A and B and Figure S6). In a different approach, we used the same probabilities in time for diploid female XX cells in a mathematical model, and obtained distributions of different cell populations that supported our findings with the stochastic simulations (Text S1, and Figure S1B).

To validate the findings, we introduced two different m values of 0.67 found in XXY triploid cells, and 0.5 found in diploid XY and tetraploid XXYY cells, keeping all other parameter values constant. The probability curves obtained with these m values resulted in a negative probability (is equal to 0) for diploid XY and tetraploid XXYY cells, as expected (Figure 4D). For XXY cells with an m = 0.67 we obtained a positive value for y, predicting initiation of XCI, albeit at an even lower level than found in XXXY tetraploid cells (Figure 4D). Using the probabilities derived with m = 0.67 we obtained simulated distributions that match well with our experimental data (Figure 4E, and Figure S7A). Raising m to 1.5, as found in females with an 47,XXX aneuploid karyotype, increases the probability to a maximum of 44% (Figure S4C and Figure 4D). Simulations using this probability curve result in a majority of cells that inactivate two X chromosomes (Figure 4E, and Figure S7B), as reported for 47,XXX human individuals. Interestingly, simulation of XXX diploid (aneuploid) cells only resulted in a 26% cell loss. This percentage is well below the 50% cell loss obtained for human individuals and viable mice with X:autosome translocations [32], [33], and does explain why mice and humans with one or more additional X chromosomes are viable. Taken together, the results show that the XCI process can be simulated using a probability curve representing the effective XCI-activator concentration in combination with a threshold level required to initiate XCI.

### Allele specific activation levels for the XCI-activator

**Figure 5:**
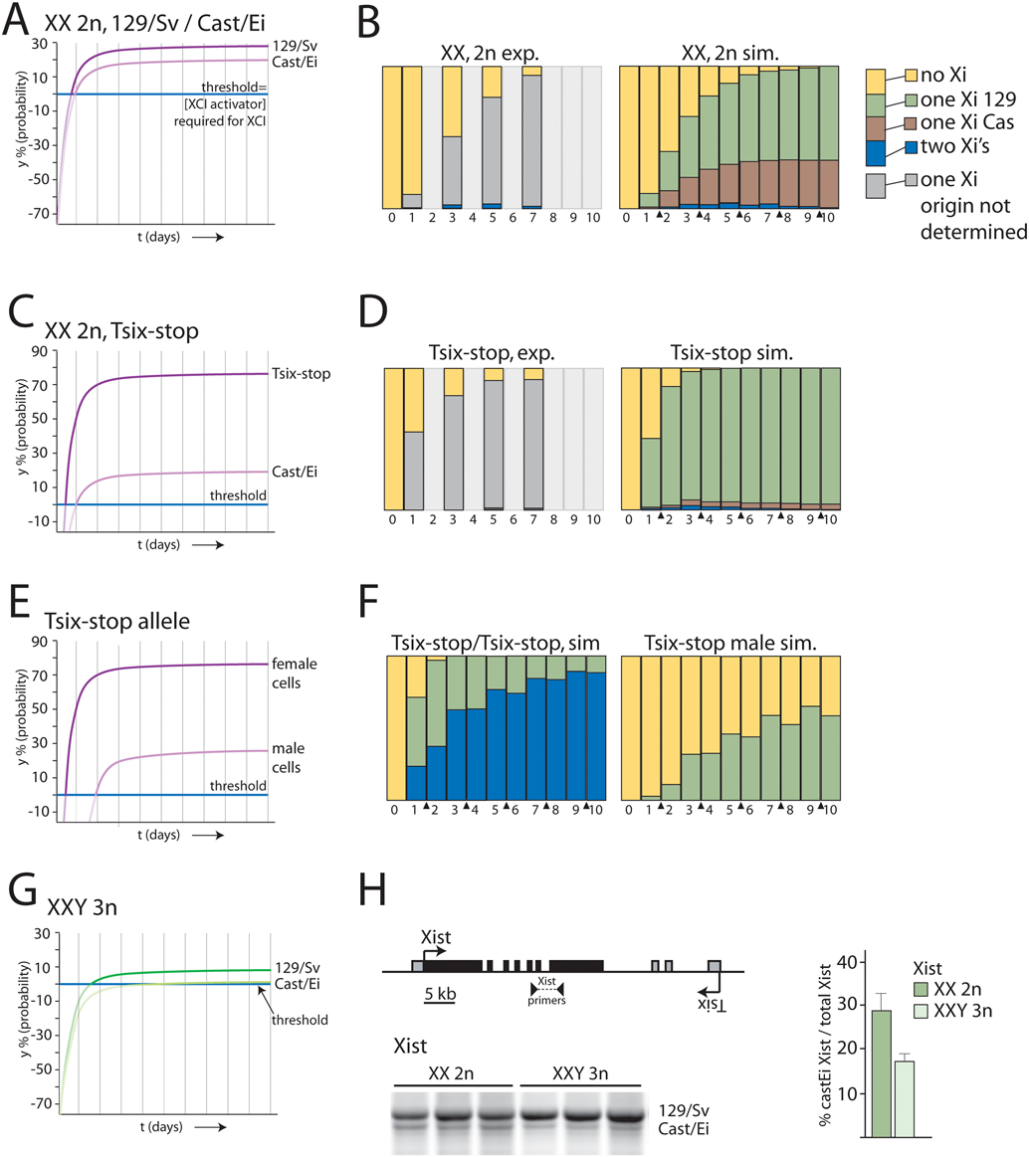
Implementing allele specific thresholds. A, B) XCI skewing can be simulated by attributing different probabilities to the two X chromosomes in female XX cells. To simulate the 30%:70% skewing of XCI observed in Cast/Ei – 129/Sv F1 mice and differentiating F1 ES cells different allele specific probabilities were applied. (B) The experimentally obtained XCI data (left panel) with differentiating XX Cast/Ei – 129/Sv F1 ES cells does not discriminate between inactivation of the Cast/Ei or 129/Sv X chromosome. Right panel shows simulations with allele specific probability curves presented in (A). C) Almost complete skewing of XCI towards the *Tsix*-stop containing 129/Sv X chromosome was simulated using allele specific probabilities for the Cast/Ei and mutated 129/Sv X chromosomes. D) Left panel shows the experimentally obtained XCI data with 2n XX heterozygous *Tsix*-stop female cells. In this experiment no discrimination was made between inactivation of the Cast/Ei and 129/Sv X chromosomes. The right panel shows simulated XCI experiments using the probability y presented in (C). E) Predicted probabilities in time for a *Tsix*-stop X chromosome in male and female ES cells. F) Left panel shows simulation experiments with homozygous female *Tsix*-stop cells. Right panel shows initiation of XCI occurring in simulation experiments with *Tsix*-stop male cells. G) Predicted probability curves for Cast/Ei and 129/Sv X chromosomes in 3n XXY ES cells. H) Schematic presentation of the *Xist* locus and the localization of the PCR primers used to determine skewing of *Xist* expression. RT PCR was performed with cDNA of 7 day differentiated 2n XX cells and 3n XXY cells, with *Xist* primers spanning intron 6, amplifying a length polymorphism present in exon 7. The average percentage, and standard deviation, of *Xist* emanating from the Cast/Ei X chromosomes relative to the total amount of *Xist* is shown in the right graph.

A stochastic model implies that different X chromosomes within one nucleus can have different probabilities to be inactivated, because X chromosome specific thresholds are determined independently. In inbred mice, the X chromosomes are genetically identical and XCI will therefore result in two evenly distributed populations of XiXa cells with one of the parental X chromosomes inactivated. However, in several F1 hybrid mice, XCI has been reported to be skewed towards one of the parental alleles. For mouse, skewing of XCI has been attributed to differences in the X controlling element (Xce), a region overlapping and extending 3′ of *Xist* [34], [35]. In cells where two X chromosomes are present with different Xce alleles, a strong Xce is associated with a lower probability to initiate XCI compared to the X chromosome harboring the weaker Xce. These reported differences in probabilities could be explained as allele specific thresholds for the XCI-activator. A more sensitive allele (weak Xce) for the XCI-activator will result in a higher probability for XCI at a certain XCI-activator concentration than a less sensitive allele (strong Xce). As a consequence, one XCI-activator concentration can result in different probabilities for different alleles in the same nucleus. *Mus musculus castaneus* (Cast/Ei) mice harbor a strong Xce in contrast to *Mus musculus* 129/SV (129/Sv) mice, which harbor a weak Xce, and in somatic tissues of Cast/Ei-129/Sv F1 female mice and differentiated F1 female ES cells, the 129/Sv X chromosome is inactivated in ∼70% of the cells (Figure 5H). Allele specific sequence differences in *Xist*, *Tsix*, and *Xite* will lead to different values for V_ys_ and/or V_yd_. As indicated above, y may represent activated XCI-activator molecules or chromatin modifications (as targets for activated XCI-activator), for which V_ys_ and V_yd_ reflect the composition and breakdown, respectively, of the complex involved in *Xist* transcriptional activation and/or *Xist* RNA mediated silencing. The values for V_ys_ and V_yd_ will depend on allelic properties of different X chromosomes, such as SNPs in the Xce region associated with allelic threshold levels to initiate XCI. We have investigated both options, using different allele specific values for V_ys_ or V_yd_. By training the stochastic simulation program, we found best-fit allele specific V_ys_ values for the 129/Sv X and Cast/Ei X alleles of 3.19 and 2.87, respectively, or V_yd_ values for the 129/Sv X and Cast/Ei X alleles of 1.05 and 1.20, respectively (Figure S4D and E). Keeping all other variables constant, these obtained values resulted in probability curves that, in stochastic simulations of XCI, generated a relative distribution of 129/Sv : Cast/Ei of 70% : 30% (Figure 5A and 5B, and Figure S8A).

Completely skewed XCI has been reported in female mice and ES cells, in which *Tsix* transcription is abrogated by deletion of *Tsix* regulatory elements or a block of *Tsix* transcription through insertion of poly-adenylation sequences. These mice and ES cells show almost completely skewed XCI towards inactivation of the *Tsix* mutant X chromosome. We have recently shown that XCI starts earlier in differentiating *Tsix* mutant ES cells compared to wild type female cells, and that by day one of differentiation already 50% of the mutant cells have initiated XCI, compared to ∼10% of the wild type cells. In the stochastic simulation, a raise in the probability for the 129/Sv X chromosome harboring the *Tsix* stop by increasing V_ys_ to 4.60 µM, results in an outcome that matched the experimental findings (Figure 5C and 5D, and Figure S4F and Figure S8B). This simulation also shows that in this case only few cells inactivate both X chromosomes, as we previously reported [10], because early during differentiation the mutant X chromosome is subject to a high probability to undergo XCI compared to the wild type X, meaning that the XCI-activator level will drop well before the second X might become a target. Using the same parameters in a simulation of homozygous female *Tsix*-stop cells we found a very high number of cells inactivating both X chromosomes (Figure 5F and Figure S9A), as was reported for differentiating female ES cells with a homozygous mutation of *Tsix* [30]. Interestingly, in *Tsix*-stop male cells with m = 0.5 we still find that y can eventually, over a period of time, reach a value above zero (Figure S4F), suggesting that the single X in these male cells will still have a probability to undergo initiation of XCI upon differentiation or development (Figure S9B). Indeed this has been reported in differentiating male ES cells with this *Tsix*-stop mutation and other mutations that abrogate *Tsix* function [36]–[39].

Although the thresholds for both Cast/Ei and 129/Sv X chromosomes are similar in F1 2–1 diploid XX cells and the triploid XXY cells that we generated for this study, the putative X-encoded XCI-activator concentration in the nucleus will be different. In the 3n XXY cells, the XCI-activator concentration will be lower than in 2n XX cells, related to the larger volume of the nucleus of triploid cells as compared to diploid cells. Hence, the allele specific threshold for the strong Xce of the Cast/Ei X chromosome in 3n XXY cells may be too high to generate a probability to start XCI (Figure 5G). To test whether this is true, we differentiated 2n XX and 3n XXY lines for 7 days (in triplo), and performed RT-PCR analysis with an *Xist* specific primer set. As predicted, we found that skewing of XCI is enhanced towards the weak Xce of the 129/Sv X chromosome in the 3n XXY cell lines (Figure 5H). We conclude that the stochastic simulation studies show that our hypothesis that the probability for an X chromosome to undergo initiation of XCI is effectuated by an X-encoded activator of XCI, above a nuclear threshold level, is feasible, at least when considered in the light of the experimental information. They also demonstrated that the probability to initiate XCI may well depend on the number of X chromosomes per nucleus, the nuclear volume related to ploidy and different thresholds for specific Xce alleles.

## Discussion

We have analyzed XCI in differentiating triploid mouse ES cells, and found that XXY cells with an X∶A ratio of 0.67 initiate XCI less frequently compared to cells with a higher X∶A ratio. Cells that do initiate XCI (XaXiY) proliferate slightly faster than XaXaY cells, and slowly accumulate in time. Simulation studies of XCI, based on a stochastic principle, indicate that XCI counting and choice can be mimicked when using a probability for an X chromosome to be inactivated, in which the probability is dependent on a nuclear XCI-activator concentration acting at differential threshold levels for X chromosomes with specific Xce alleles.

### Triploid ES cells and the need for speed

In this study, we have generated triploid mouse ES cells by PEG mediated fusion of diploid ES cells with haploid round spermatids. Interestingly, we could only generate triploid ES cells with a XXY karyotype, in which the Y chromosome was donated by the round spermatid. The fact that we could not generate an ES cell line with the same XXY karyotype by fusion of a male ES cell with a round spermatid donating an X chromosome indicates that the presence of a spermatid derived X chromosome results in a triploid cell that is not viable. This difference could be the consequence of epigenetic interference with transcription of the X chromosome from spermatids, which hampered the viability of our triploid cells. However, such an effect has never been reported in mice [40]. Epigenetic modification of the X in spermatids might be a consequence of meiotic sex chromosome inactivation (MSCI) [25], that is overcome in normal sperm development and fertilization, but cannot be reversed by the ES cell. Indeed, fusion experiments with round spermatids harboring an X-linked *GFP* transgene indicate that the spermatidal X chromosome is not reactivated by the ES cell. In contrast, fusion of ES cells with somatic XaXi diploid cells results in proper reactivation of the inactive X chromosome [27]. Therefore, the apparent absence of spermatidal X reactivation in our triploid XXY and XXX ES cells indicates the presence of epigenetic differences laid down on an Xi during the MSCI and the XCI processes. The Y chromosome is also subject to MSCI, but fusion of XX diploid ES cells with a round spermatid containing a Y chromosome does result in viable 3n ES cell lines. This shows that the spermatidal autosomes do not affect the viability. The Y chromosome has little gene content, compared to the large X chromosome, such that possible epigenetic modification of the Y chromosomes by MSCI may not impact on the outcome of the fusion process.

The absence of triploid XYY ES cells can be attributed to these cells having an X∶A ratio of 0.33, which is probably lower than required for normal viability and growth for ES cells. Lethality due to an elevated level of Y chromosome transcripts is not likely, in view of the viability of 47,XYY aneuploid male individuals. Although XYY triploid embryos have been observed to occur in mouse and human, the observed frequencies are much lower than expected [41], [42]. Interestingly, for differentiating mouse triploid XXY ES cells we find many cells with a single Xa, indicating that an X chromosome under-dosage problem, of one active X per triploid genome, plays a role in particular in undifferentiated ES cells or during early embryonic development. Moreover, after 10 days of EB differentiation of these XXY triploid ES cells, we found an increase in the relative number of XaXiY cells, making up 41% of the total cell population. This indicates that XaXiY is the inactivation pattern that results in a cell with the preferred dosage of X-linked genes. This observation is supported by previous *in vivo* experiments, examining XCI in mouse XXY and XXX triploid 10 dpc (days *post coitum*) embryos, which showed that 83% of the cells were XaXiY, and 92% of the cells were XaXiXi, respectively [21]. Therefore, we conclude that mouse triploid cells preferably keep only one of their X chromosomes active.

The present observation that after three days of ES cell differentiation 3–4% of XXY triploid ES cells have started XCI, provides additional evidence for the hypothesis that the X∶A ratio indeed determines the probability to initiate XCI. Our studies also show that the XCI initiation rate for the differentiating XXY triploid ES cells is too low to allow all cells to inactivate one X chromosome within the time span where XCI can be initiated. These cells cannot meet the need for speed.

### XCI counting and initiation

The finding that the probability to initiate XCI is proportional to the X∶A ratio suggests the presence of an X-linked gene encoding an XCI-activator, which itself is transcriptionally inactivated during the XCI process. During differentiation or development, the nuclear concentration of this XCI-activator will increase and reach a threshold level required to generate a probability to initiate XCI. In cells with a relatively high X∶A ratio, the XCI-activator concentration will reach the threshold level at an earlier time point and will plateau at a higher level, and therefore generate a higher probability, compared to cells with a lower X:A.

Silencing of one of the XCI-activator genes in female cells will lead to a drop in the XCI-activator level equal to that found in male cells, which is not sufficient to initiate XCI on the remaining X. Nevertheless, *Xist* remains expressed on the Xi because its negative regulator *Tsix* is also silenced in *cis*, allowing a lower XCI-activator concentration to maintain *Xist* expression. Persistent expression of *Tsix* on the Xa results in silencing of *Xist* on that chromosome. In female mice and cell lines with only one functional copy of *Xist*, XCI will only be initiated on the wild type X chromosome. If *Tsix* is intact on the other X chromosome the non functional *Xist* gene is silenced in *cis* [43]. However, in female cell lines heterozygous for a single allelic region containing both a non-functional *Xist* and *Tsix* gene, the *Xist* promoter of the mutated allele will not be silenced. This explains the reported persistent expression of non-functional *Xist* from the Xa, and the finding that this mutant *Xist* promoter adopts the same chromatin configuration as found for the wild type *Xist* promoter on the Xi [44].

A stochastic model for XCI, involving an X-encoded XCI-activator, assumes that the nuclear volume is directly proportional to the ploidy, which in mice is indeed the case [45], [46]. We found that triploid XXY ES cells, and tetraploid XXXY and XXXX ES cells showed a significant difference in the number of cells that initiated XCI after three days of differentiation, supporting the presence of an XCI-activator. Moreover, tetraploid XXXX cells have initiated XCI after three days of differentiation more effectively than diploid XX cells, despite a similar XCI-activator concentration (number of X chromosomes per nuclear volume). We attribute this difference to the different number of X chromosomes, that each have a probability to initiate XCI. This is supported by our simulations, that also show a faster increase of tetraploid XXXX cells with one or more Xi's than the rate of appearance of diploid XX cells with an Xi.

The counting and initiation phase of XCI has also been explained by the presence of an autosomally encoded blocking factor or nuclear entity, of which one dose or one functional unit is present in the diploid nucleus, preventing inactivation of one X chromosome. We advocate that our current and previous findings do not support a blocking factor model for the XCI counting and initiation process. Examination of XCI after a 3-day differentiation period of the different tetraploid cell lines showed that XCI counting works properly in XXYY tetraploid cells, which only sporadically initiate XCI. Nevertheless, for the XXXY and XXXX tetraploid cell lines, we found many cells that initiated XCI on the wrong number of X chromosomes, more than one in XXXY and more than two in XXXX tetraploid cells [10]. A blocking factor model cannot explain these results because the two functional units of blocking factor (present in tetraploid cells) that properly block XCI on both X chromosomes in XXYY cells should have done the same in XXXY and XXXX cells, which is not the case. Nevertheless, the actual results could be explained if the blocking factor is assembled out of a limiting amount of molecules as predicted by the symmetry-breaking model [47], which is used up with an increasing number of X chromosomes, but a comparison of our results of diploid XX cells with triploid XXY cells argues against this possibility. We found a much lower number of triploid XXY cells compared to diploid XX cells that initiated XCI at day 3, despite the fact that the concentration of the molecules making up the blocking factor would be the same in both cell lines.

### Cellular population dynamics of XCI

The present simulation studies of XCI indicate that the XCI counting and initiation process can be simulated by inclusion of relatively few variable parameters. First, there is a probability to initiate XCI for any individual X chromosome. Second, specific Xce alleles respond to different nuclear threshold levels of an XCI-activator. This is all that is required to explain the initiation of XCI. The simulations only tested whether a stochastic model for XCI could explain the available and new experimental data. Other models explaining the initiation phase of XCI, including the blocking factor, symmetry breaking, and transvection models, hypothesize that XCI is directed by a mutual exclusive choice process [48]. Unfortunately, this situation could not be simulated in our program. Nevertheless, the simulations based on a stochastic model make predictions, some of which we have thoroughly tested and other predictions that await further analysis.

In the computer simulations, we have used ten XCI choice rounds over a 10 day differentiation period. However, *in vivo* the number of choice rounds may be less than ten, resulting in more cells with too many Xa's, which would be selected against. This is supported by observations made in female embryos that show a significant number of cells with two Xa's after completion of the X inactivation process [21], [49]. Our simulations also predict less cells with too many Xi's than we detected *in vivo*, especially for the 4n XXXX and XXXY cells. This can be explained, if initiation of XCI on the right number of X chromosomes does not result in an immediate drop of the XCI-activator level below the threshold, so that XCI can still be initiated on additional chromosomes until turnover of the XCI-activator has resulted in a drop below the threshold level. We have not incorporated this possibility in our simulation program.

With regard to embryo development, it is interesting that simulations with 2n XX ES cells indicate that the cell number in female diploid XX embryos will be significantly reduced by about 12% when compared to male diploid XY embryos (Figure S5, blue box). This is in the range of reported size differences between female and male embryos around the time XCI has been completed, and before hormonal cues start to influence growth of the embryo [50]. Therefore, this reported *in vivo* size difference could be explained by female specific cell loss as a by-product of the X inactivation process. Furthermore, for female homozygous *Tsix*-stop cells, our simulation showed that almost all the cells are lost during the XCI process. In male ES cells the reduction in expected cell number is 88%. This may explain the reported sex-ratio distortion in homozygous ΔCpG *Tsix* knockout mice [51]. However, the high loss of cells in our simulations of male and female embryos with a homo/hemizygous *Tsix*-stop mutation indicates that these mice will most likely not be viable. Interestingly, viable mice, albeit at a lower mendalian ratio, have been reported with a homo/hemizygous ΔCpG *Tsix* knockout allele suggesting that the probability to initiate XCI for this allele is lower than for the *Tsix*–stop allele used in our simulations [51]. This indicates that the ΔCpG *Tsix* allele is a partial knockout of *Tsix*, which is supported by *in vivo* studies showing that a hemizygous *Tsix*-stop allele results in a non-viable phenotype, in contrast to the hemizygous ΔCpG *Tsix* mice that are viable and breed [51], [52]. Also, other mutations of *Tsix* result in activation of *Xis*t in male cells upon ES cell differentiation, in contrast to male cells with a ΔCpG *Tsix* mutation that do not show initiation of XCI [36]–[39].

The XCI-activator has not been identified yet. However, several lines of evidence indicate that it acts through *Xist*, and could be a protein or RNA involved in activation and/or stabilization of *Xist* [10]. Studies with different *Tsix* mutant cell lines suggest that, in mice, *Tsix* plays a crucial role in determining the XCI-activator level required for generating a probability to initiate XCI by suppression of *Xist*. In addition, chromatin modifications of the *Xist* promoter may also play a role in determining the threshold, which might be even more relevant in human were the presence and function of *TSIX* are still speculative. Identification and characterization of the XCI-activator, and factors involved in setting up the threshold, will be of crucial importance for a better understanding of the initiation phase of XCI.

## Materials and Methods

### Culture and differentiation of ES cells

ES cells were cultured in DMEM supplemented with 15% heat inactivated foetal calf serum, 100 U ml^−1^ penicillin, 100 mg ml^−1^ streptomycin, non-essential amino acids, 1000 U/ml leukaemia inhibitory factor (LIF) and 0,1 mM β-mercaptoethanol. ES cells were grown on a layer of male mouse embryonic fibroblast (MEF) feeder cells. To induce differentiation into EBs, ES cells were pre-plated for 60 minutes and non-adherent ES cells were transferred to non-gelatinized bacterial culture dishes without feeder cells in differentiation medium, IMDM Glutamax, 15% heat inactivated foetal calf serum, 50 µg/ml ascorbic acid, 100 U ml^−1^ penicillin, 100 mg ml^−1^ streptomycin, 37.8 µl/l monothioglycerol.

### Mice and staput isolation of round spermatids

All animals were treated in accordance with guidelines of the Erasmus MC, Rotterdam, the Netherlands. Testes from two *Ube2a* homozygous mutant mice and two *Ube2b* heterozygous mutant mice were excised and decapsulated to remove the tunica albuginea. Decapsulated testes were pooled in 20 ml PBS (140 mM NaCL, 3 mM KCl, 1.5 mM KH_2_PO, 8 mM NaH_2_PO_4_)/1.1 mM Ca^2+^/0.5 mM Mg^2+^/12 mM lactate (Sigma-Aldrich) of 34°C, containing 10 mg hyaluronidase (from ovine testes, Roche-Diagnostics), 20 mg trypsin (from bovine pancreas, Roche-Diagnostics) and 20 mg collagenase A (Roche-Diagnostics). Testes were shaken for 20 minutes at 90 rpm with 10 mm amplitude to release seminiferous tubuli from interstitial cells. Tubuli were collected by centrifugation for 3 minutes at 2000 rpm and resuspended in 34°C PBS/12 mM lactate. After shaking 10 minutes at 120 rpm with 10 mm amplitude to release germinal cells from the tubuli, tubuli remnants were removed. Germinal cells were collected by centrifugation and resuspended in 34°C PBS/1.1 mM Ca^2+^/0.5 mM Mg^2+^/12 mM lactate. The cell suspension was filtrated using a 60 µm filtration cloth. Germinal cells were collected by centrifugation and resuspended in 50 ml PBS/1.1 mM Ca^2+^/0.5 mM Mg^2+^/12 mM lactate/0.5% w/v BSA. Cells were separated by sedimentation velocity at unit gravity in a 1–4% w/v BSA gradient at room temperature. First 20 ml PBS/1,1 mM Ca^2+^/0.5 mM Mg^2+^/12 mM lactate was bottom-loaded in a chamber, followed by 50 ml cell suspension. A BSA gradient was created by loading a total of 500 ml of 1%, 2% and 4% w/v BSA in PBS. Cells were allowed to sediment for 2 hours. The chamber was emptied in 8 ml fractions using a fraction collector, and fractions containing peak amounts of cells were identified using a 340 nm UV light source. Fractions containing round spermatids were pooled, collected by centrifugation and resuspended in PBS/1.1 mM Ca^2+^/0.5 mM Mg^2+^/12 mM lactate. Purity of round spermatid preparations derived by this procedure were shown to be >90%, as determined by microscopic analysis of an aliquot of purified cells fixed in Bouins' fixative on glass slides [53].

### Fusion experiments

*Mus musculus* castaneus/129/Sv F1 (F1-2 1) female and C57Bl6/129/Sv (V6.5) male ES cell-lines were separated from MEF feeder cells by trypsinizing and preplating for 45 minutes on uncoated culture dishes. PEG1500 fusion was performed according to the manufacturer's instructions (Invitrogen). Briefly, 4·10^6^ cells were combined with 4·10^6^ round spermatids in DMEM. After centrifugation cells were resuspended in 300 µl 50% v/v PEG1500 and incubated for 2 minutes at 37°C under continuous stirring. The mixture was gradually diluted with serum containing medium and plated on drug-resistant MEF feeder cells. After 24 hours medium was replaced with medium containing 0.3 µg/ml neomycin and 2 µg/ml puromycin. After nine days, individual ES cell colonies were picked, trypsinized and plated on individual culture dishes in neomycin and puromycin containing medium.

### Cell cycle block

ES cells were EB differentiated for one or two days and then blocked in the cell cycle by adding 0.75 mM mimosine, 12 µl/ml colcemid (KaryoMax, Gibco) or 2100centiGray γ-irradiation. Cells were fixed one day after applying the cell cycle block.

### Karyotyping

ES cells were blocked in metaphase by incubation in medium containing 0.12 µg/ml colcemid for 1 hour. Cells were trypsinized and resuspended in 5 ml 0.075 M KCl at 37°C, collected and resuspended in 0.0625M KCl/12.5% methanol/4.17% acetic acid. Cells were fixed by washing three times in 75% methanol/25% acetic acid and stored in 200 µl at 4°C. The fixed cell suspension was spotted on ethanol cleaned slides and air dried. For determining the total number of chromosomes slides were mounted with 20 µl Dapi vectashield.

To determine the number of X chromosomes, slides were denatured by a three minute incubation at 80°C in 100 µl 50% formamide/2×SSC/10 mM phosphate buffer. Subsequently slides were dehydrated, and hybridized overnight at 37°C with a Cy3 labelled X-paint probe (Cambio). After hybridization, slides were washed once with 2×SSC at 45°C, three times with 2×SSC/50% formamide at 45°C and two times with PBS. Slides were dehydrated through ethanol steps (70%, 90% and 100%) air-dried and mounted with 20 µl dapi vectashield. For determining the number of Y chromosomes, Y-chromosome paint (Cambio) was applied, following the same protocol as for the X chromosome paint.

### RNA FISH analysis

One day prior to fixation, non-adherent EBs were trypsinized and differentiated ES cells were grown on gelatin-coated cover slips. Cells were rinsed once with PBS and permeabilized by successive incubation in cytoskeletal buffer (100 mM NaCl, 300 mM sucrose, 3 µM MgCl_2_, 10 mM PIPES pH 6.8 in H_2_O) for 30 seconds, cytoskeletal buffer containing detergent (0.5% triton X-100, 100 mM NaCl, 300 mM sucrose, 3 µM MgCl_2_, 10 mM PIPES pH 6.8 in H_2_O) for 2 minutes and cytoskeletal buffer for 30 seconds. Cells were fixed in 4% paraformaldehyde/PBS for 10 minutes, rinsed three times with 70% ethanol and stored in 70% ethanol at 4°C.

The *Xist* probe was a digoxygenin labelled 5.5 kb cDNA sequence [10]. To suppress repetitive sequences 25 µg/ml mouse Cot1 DNA was added and probe mixture was incubated at 95°C for 5 minutes and at 37°C for 45 minutes. After overnight hybridization at 37°C, slides were washed in 2×SSC at 37°C for 5 minutes, and three times in 50% formamide/2×SSC at 37°C for 10 minutes. Probe detection was performed at room temperature. Detection was with a sheep anti-digoxigenin antibody (Roche diagnosics), followed by a FITC labelled rabbit anti-sheep antibody (Jackson labs) and a FITC labelled goat anti-rabbit antibody (Jackson labs), each for 30 minutes, in 100 mM Tris pH 7.5/saline/Tween, BSA. After detection cover slips were dehydrated and mounted on a slide in Vectashield and DAPI to counter stain DNA. To determine the number of inactive X chromosomes in a cell, a non-overlapped intact nucleus was selected, and the number of *Xist* clouds were scored.

### BrdU analysis

For BrdU analysis, differentiated ES cells of trypsinized non-adherent EBs were grown on gelatin-coated cover slips in the presence of 20 µM BrdU, and fixed as described in the RNA FISH section. Cover slips were dehydrated, air-dried and denatured in 70% formamid/2×SSC/50 mM phosphate for 3 minutes at 85°C. Coverslips were washed in ice cold 70% ethanol and through 70%, 90% and 100% ethanol washes and air dried after which the *Xist* probe was applied. Detection of *Xist* RNA was as described in the previous section, detection of BrdU was performed with a mouse monoclonal BrdU antibody (DAKO), followed by a rhodamin labelled donkey anti-mouse antibody (Jackson labs), 30 minutes incubation each.

To determine the number of BrdU labelled cells for the XaXaY and XaXiY cell populations, first a microscope field was selected, containing one or more intact nuclei with an Xist cloud. Within this field, the number of cells containing an Xist cloud with negative or positive BrdU staining was determined. Subsequently this was also done for all cells without an Xist cloud in the same microscopic field.

### DNA FISH analysis

For DNA FISH, cells were fixed as for RNA FISH, and pretreated for 4 min with 0.5% pepsin in 10 mM HCl at 37°C, post fixed for 5 minutes in 4% paraformaldehyde/PBS, washed twice with PBS, and dehydrated prior to denaturation. Denaturation of target sequences was as described in the BrdU analysis section. Cover slips were incubated with a combination of two biotin-labelled BACs (CT7-155J2 and CT7-474E4) at 37°C overnight. BACs were detected using mouse anti-biotin (Roche diagnostics) and donkey anti-mouse antibodies (Jackson labs) as described for RNA FISH. To determine the number of X chromosomes, non-overlapping nuclei were selected and the number of signals per nucleus was determined.

### Genotyping and RT PCR analysis

For genotyping the mutant Ube2b allele was amplified with primers CTTTACGGTATCGCCGCTCCCGAT, TTGAAATCCCGCATGAGC, and CGGAGGGAGACGTCATTG. For RT-PCR RNA was isolated with Trizol reagent, treated with RNAse free DNAse and reverse transcribed (all Invitrogen). *Xist* RNA was amplified with primers ACTGGGTCTTCAGCGTGA, and GGGAATAGGTAAGACACACTG spanning intron 6, which amplify a length polymorphism in exon 7 (129/Sv fragment is 888 bp, Cast/Ei fragment is 845 bp).

### Stochastic simulations

Stochastic simulations were performed in a SQL based program (the source code can be found in the Text S1), using 10 Z-stacks, and 100 starting cells. The program allows the use of different probabilities in time, a different number of X chromosomes per cell, and a different rate of cell division depending on the number of Xi's.

## Supporting Information

Text S1 (0.12 MB DOC)

Figure S1 Mathematical computation of cell populations A) The panels show the mathematical computation the XaXa, XaXi and XiXi populations with a 5%, 10%, 20%, 30%, and 40% fixed probability per X chromosome. The different bar-graphs show the relative distribution of the three different cell types (XaXa = green, XiXa = red, XiXi = blue), in a 22 day differentiation experiment. B) This panel shows the mathematical computation the XaXa, XaXi and XiXi populations with a changing probability for m = 1 presented in figure 4B.
(3.11 MB TIF)

Figure S2 Stochastic simulations with a fixed probability ranging from 5% to 30% A, B and C) Results of the stochastic simulations using a fixed probability ranging from 5% to 20%. The average of five independent runs is highlighted in green. The expected number of XiXa cells and the experimentally obtained number of XiXa cells is highlighted in blue.
(3.71 MB TIF)

Figure S3 Stochastic simulations with a fixed probability ranging from 30% to 50% A, B and C) Results of the stochastic simulations using a fixed probability ranging from 30% to 50%. The average of five independent runs is highlighted in green. The expected number of XiXa cells and the experimentally obtained number of XiXa cells is highlighted in blue.
(3.62 MB TIF)

Figure S4 Calculation of the probability y A, B) This figure shows (B) the XCI-activator concentration in a nucleus with a different X∶A ratio (m), based on values for the different variables given in (A). (C) The probability y was determined for cells with a different number of sex chromosomes and/or ploidy. D, E, F) Show the allele specific probability y in time with different Vyd or Vys values in wild type (D, E) and Tsix-stop cells (F), used in our simulation experiments.
(1.84 MB TIF)

Figure S5 Stochastic simulation of XCI in diploid XX and tetraploid XXXX cells A, B) Results of the stochastic simulations using the probability curves shown in Figure 4B for diploid XX (A) and tetraploid XXXX cells (B). The average of five independent runs is highlighted in green. The expected number of XiXa and XiXiXaXa cells and the experimentally obtained number of XiXa and XiXiXaXa cells from the diploid and tetraploid cells respectively are highlighted in blue.
(3.07 MB TIF)

Figure S6 Stochastic simulation of XCI in tetraploid XXXY cells Results of the stochastic simulations using the probability curves shown in Figure 4B for tetraploid XXXY cells. The average of five independent runs is highlighted in green. The expected and obtained number of tetraploid XiXaXaY cells are highlighted in blue.
(1.77 MB TIF)

Figure S7 Stochastic simulation of XCI in triploid XXY and diploid XXX cells Results of the stochastic simulations using different probability curves presented in Figure 4D for triploid XXY cells (A) and diploid XXX cells (B). The average of five independent runs is highlighted in green. Except for the triploid XXY cells, the expected and obtained number of viable cells is highlighted in blue.
(2.70 MB TIF)

Figure S8 Stochastic simulation of XCI in diploid cells with allele specific probabilities A, B) Results of stochastic simulations using the X∶A ratio of 1, and allele specific probabilities indicated in Figure 5A (A) and 5C (B). (A) shows the simulation of F1 female Cast/Ei 129/Sv cells, (B) heterozygous female Tsix-stop cells. The average of five independent runs is highlighted in green. The expected and obtained number of viable cells are highlighted in blue.
(2.68 MB TIF)

Figure S9 Stochastic simulation of XCI in female and male cells with a Tsix-stop allele A, B) Results of stochastic simulations using the X∶A ratio of 1, and allele specific probabilities indicated in Figure 5E. Simulation experiments with homozygous female (A) and hemizygous male (B) Tsix-stop alleles. The average of five independent runs is highlighted in green. The expected and obtained number of viable cells are highlighted in blue.
(2.46 MB TIF)
