# Supplementary material for: The Probability to Initiate X Chromosome Inactivation Is Determined by the X to Autosomal Ratio and X Chromosome Specific Allelic Properties
Source: PLoS One. 2009 May 19;4(5):e5616. doi: 10.1371/journal.pone.0005616 (PMC2680018; doi:10.1371/journal.pone.0005616)
Supplement: Text S1 — (0.12 MB DOC) [file pone.0005616.s001.doc]

**Supplementary data**

***Mathematical calculation, using fixed and changing probabilities***

**Cell division and choice rounds**

*C_m_*

d1

d2

*C_5_*

*C_6_*

*C_3_*

*C_4_*

d..m

*q*

*q*

*q*

*q*

*q*

*q*

*C_1_*

*C_2_*

XaXa= n

XaXi = 0

XiXI = 0

*C*

Figure: A schematic representation of the distribution of the different cell populations after a number of cell divisions and X inactivation rounds. *n* number of cells enter into the X inactivation process with a distribution of XaXa = n, XaXi = 0, and 0 XiXi =0. As these n numbers of cells undergo a differentiation process by choosing to inactivate an X chromosome (c_1_ ,c_2 …_) with a probability q at any cell division (d_1_), their distribution can be calculated by using these variables.

*Abbreviations*

n = the number of cells at the beginning of the whole event

d = number of cell divisions

c = number of choice rounds

p = the probability for an X chromosome to remain active

q = the probability for an X chromosome to be inactivated

XaXa = The number of cells with two active X chromosomes

XaXi = The number of cells that inactivated one of the two X chromosomes

XiXi = The number of cells that inactivated both X chromosomes

d_o_c_1_ = 0 cell divisions and 1 choice round of X chromosome inactivation

d_1_c_3_ = 1 cell division and 3 choice rounds of X chromosome inactivation

*XaXa cell population*

By assuming that an X chromosome in a cell remains active with a certain fixed probability *p* at every choice round, the number of XaXa cells after *c* number of choice rounds and *d* number of divisions can be calculated as:

d_0_c_1_ = P^2^(n)

d_0_c_2_ = P^2^(P^2^n) = p^4^ (n)

d_1_c_3_ = P^2^ (2 (P^4^n)) = 2p^6^ (n)

d_1_c_4_ = P^2^ (2(P^6^n)) = 2p^8^ (n)

d_2_c_5_ = P^2^ (2^2^(P^8^n)) = 2^2^p^10^ (n)

d_2_c_6_ = P^2^ (2^2^(P^10^n)) = 2^2^p^12^ (n)

From the above distributions we can derive that the amount of XaXa cells at any point is:

XaXa = 2^d^(P^2c^)(n)

If this probability p is changing in time then,

d_0_c_1_ = P_1_^2^(n)

d_0_c_2_ = P_2_^2^(P_1_^2^n) = (P_2_^2^P_1_^2^ )(n)

d_1_c_3_ = P_3_^2^ (2( P_2_^2^P_1_^2^ n)) = 2 (P_3_^2^ P_2_^2^P_1_^2^ )(n)

d_1_c_4_ = P_4_^2^ (2 P_3_^2^ P_2_^2^P_1_^2^ )(n) = 2 (P_4_^2^ P_3_^2^ P_2_^2^P_1_^2^ )(n)

d_2_c_5_ = P_5_^2^ 2(2 (P_4_^2^ P_3_^2^ P_2_^2^P_1_^2^ ))(n) = 2^2^(P_5_^2^P_4_^2^ P_3_^2^ P_2_^2^P_1_^2^ )(n)

d_2_c6 = P_6_^2^2^2^(P_5_^2^P_4_^2^ P_3_^2^ P_2_^2^P_1_^2^ )(n) = 2^2^(P_6_^2^P_5_^2^P_4_^2^ P_3_^2^ P_2_^2^P_1_^2^ )(n)

From this the number of XaXa cells after *c* number of choice rounds and *d* number of cell divisions is:

XaXa = 2^d^ (P_1_^2^P^2^_2…_P_c_^2^)( n)

*XiXi cell population*

The XiXi cell population can also be calculated as

d_0_c_1_ = q^2^(n)

d_0_c_2_ = q^2^P^2^(n)

d_1_c_3_ = q^2^(2(P^4^(n))

d_1_c_4_ = q^2^(2(P^6^(n))

d_2_c_5_ = q^2^ (2^2^(P^8^n))

d_2_c_6_ = q^2^ (2^2^(P^10^n))

After *c* choice rounds and *d* division rounds, the number of XiXi cell can be calculated by:

XiXi = 2^d^q^2^ (p^2(c-1)^)

The total amount of XiXi cells that are produced after *c* number of choice rounds and d number of divisions can be calculated by adding the amount of XiXi cells that are produced at each successive step:

$\sum XiXi_{div}$ = (2^d^q^2^ (p^2(c-1)^))n

With a non-dividing XiXi population this equation will be:

$\sum XiXi_{non-div}$ = (q^2^ (2^d^ p^2(c-1)^))n

In the case of a changing probability, where the probability to inactivate an X chromosome changes over time , the dividing XiXi cell population can be calculated by

d_0_c_1_ = q_1_^2^(n)

d_0_c_2_ = q_2_^2^P_1_^2^(n)

d_1_c_3_ = q_3_^2^(2(P_1_^2^ P_2_^2^ (n)))

d_1_c_4_ = q_4_^2^(2(P_1_^2^ P_2_^2^ P_3_^2^ (n)))

d_2_c_5_ = q_5_^2^(2^2^(P_1_^2^ P_2_^2^ P_3_^2^ P_4_^2^ (n)))

d_2_c_6_ = q_6_^2^(2^2^(P_1_^2^ P_2_^2^ P_3_^2^ P_4_^2^ P_5_^2^ (n)))

Form this we can derive that, the number of XiXi after c choice rounds and d number of divisions

XiXi = 2^d^ (q_c_^2^) (P_1_^2^ P_2_^2^ P_3_^2^ … P_(c-1)_^2^)

The total number of XiXi cells after *c* choice rounds and *d* number of divisions can be calculated by adding the XiXi cells that are generated at each step

$\sum XiXi_{div}$ = 2^d^ (q_c_^2^) (P_1_^2^ P_2_^2^ P_3_^2^ … P_(c-1)_^2^)n

With a non-dividing XiXi population this equation will be:

$\sum XiXi_{non-div}$ = (q_c_^2^) (2^d^ P_1_^2^ P_2_^2^ P_3_^2^ … P_(c-1)_^2^)n

*XaXi cell population*

The amount of XaXi cells can be calculated based on the fact that the total number of cells in the system at any point is equal to the n number of cells produced from n number of starting cells undergoing *c* number of X inactivation rounds and *d* number of cell divisions.

The total number of cells in a system at any given point is

N _(c,d)_ = XaXa_(c,d)_ + XaXi_(c,d)_ + $\sum XiXi_{div}$ _(c,d)_

The number of XaXi cells can be calculated by

XaXi = N _(c,d)_ -(XaXa_(c,d)_ + _(c,d)_)

***Calculation of the XCI-activator concentration after the start of XCI***

When one X chromosome is inactivated at t=t_i_, the further time integration gives:

In other words, the activator concentration is obtained by the same equation as before except that a term corresponding to the results of the activity of one chromosome should be subtracted. With 2 X chromosomes inactivated, a further such correction should be carried out form the time of inactivation of the second chromosome onwards, but we shall not be considering this case.

***Source Code for the stochastic simulation program***

<?php

/*******************************************************************************************/
/*
/*
/*******************************************************************************************/

require_once LIBRARY_ROOT.'pager/Pager.php';

/**
* Experiment Page
*
*/
class Page extends Pager
{
  /**
   * Experiment data
   * @var Record
   */
  private $experiment;

  /**
   * Inactivation Arr
   * @var array Record
   */
  private $inactivation_arr = array();

  /**
   * Inactivation Curve
   * @var Record
   */
  private $inactivation_curve;

  /**
   * Duplication Arr
   * @var array Record
   */
  private $duplication_arr = array();

  /**
   * The current experiment mode
   * @var int
   */
  private static $mode;

  /**
   * The current step number in the experiment
   * @var int
   */
  private static $step_nr;

  /**
   * Constructor
   *
   * @access public
   * @return string HTML
   */
  public function __construct()
  {
      parent::__construct();

      // Get experiment
      if (!$experiment_id = PageData::getFromUri(1)) return;
      if (!$this->experiment = Record::getRecord('xci_experiments', array('id'=>$experiment_id))) return;

      // get the linked records
      $linked_record_arr = CMSRecord::getLinkedRecords('xci_experiment_inactivation', $this->experiment);
      foreach($linked_record_arr as $linked_record) {
          $this->inactivation_arr[$linked_record->getXci()] = $linked_record;
      }
      $linked_record_arr = CMSRecord::getLinkedRecords('xci_experiment_duplication', $this->experiment);
      foreach($linked_record_arr as $linked_record) {
          $this->duplication_arr[$linked_record->getXca()] = $linked_record;
      }
      // sort array by key ASC
      ksort($this->duplication_arr);

      // get the inactivation curve
      if ($this->experiment->getInactivationCurve()) {
          $this->inactivation_curve = Record::getRecord('xci_experiment_inactivation_curves', array('id'=>$this->experiment->getInactivationCurve()));
      }

      // constructor guard, the number of inactivation records must be the same as the duplication records
      // check for inactivation curve
      if ((count($this->inactivation_arr) != count($this->duplication_arr)) ||
          (is_null($this->inactivation_curve))) {
          return;
      }

      // get experiment mode
      if (PageData::getFromUri(2) == 'start') {
          self::$mode = 1;
      } elseif (PageData::getFromUri(2) == 'start2') {
          self::$mode = 2;
      }

      // start experiment if mode found
      if (self::$mode) {
          $this->startExperiment();
      }
  }

  /**
   * Content getter
   *
   * @access public
   * @return string HTML
   */
  public function getCenterContent()
  {
      $html = '';

      $html .= '<h1>X Chromosome Inactivation</h1>';

      // constructor guard, the number of inactivation records must be the same as the duplication records
      if (count($this->inactivation_arr) != count($this->duplication_arr)) {
          $html .= '<h2>Inactivation / Duplication record count rate mismatch, please check.</h2>';
      }


      if ($this->experiment) {
          $html .= '<h2>'. $this->experiment->getName('text') .'</h2>';

          $html .= '
              <p>
                  <a id="mode1" class="start" title="start experiment mode I" href="'. $this->section->uri .'/'. $this->experiment->getId() .'/start">
                      &raquo; start mode I <br />
                      <small>inactivation curve per X, duplication rate per state</small>
                  </a>
              </p>';

          $html .= $this->renderStateGraphTable(1);

          $html .= '
              <p>
                  <a id="mode2" class="start"  title="start experiment mode II" href="'. $this->section->uri .'/'. $this->experiment->getId() .'/start2">
                      &raquo; start mode II <br />
                      <small>single inactivation curve, threshold per X, duplication rate per state, downscale per state</small>
                  </a>
              </p>';

          $html .= $this->renderStateGraphTable(2);
      }

      return $html;
  }

  /**
   * Render the state graph/table
   *
   * @access public
   * @return string HTML
   */
  public function renderStateGraphTable($mode=1)
  {
      $html = '';

      // get the data in a nice array
      $exp_data_arr = $this->getExperimentDataArr($mode);

      // render the table rows
      $state_graph_arr = array();
      foreach (range(0, $this->experiment->getNumberOfDays()) as $step_nr) {

          // skippit if this step is not logged
          if (!isset($exp_data_arr[$step_nr])) continue;

          // get the logged state at this step
          $state_arr = $exp_data_arr[$step_nr];

          // render the state
          $state_graph_arr[$step_nr] = '';
          $data_arr['height'] = 0;
          $z_index = pow(2, $this->getNumberOfXc()) + 10;
          foreach (range(0, $this->getNumberOfXc()) as $state) {

              // render the state types
              if (!isset($state_arr[$state])) continue;

              // default data for this state in this step
              $data_arr['percentage'] = 0;
              $data_arr['cell_count'] = 0;

              foreach ($state_arr[$state] as $state_type=>$state_type_arr) {

                  $data_arr['height']     += $state_type_arr['height'];
                  $data_arr['percentage'] = $state_type_arr['percentage'];
                  $data_arr['cell_count'] = $state_type_arr['cell_count'];

                  // generate state-count div for this state in this step
                  //$z_index = $this->getNumberOfXc() + $state_type + 10;
                  $state_graph_arr[$step_nr] .= '
                      <div class="state-count state-'. $state .'"                            style="height: '. round($data_arr['height']) .'px; z-index:'. $z_index .';"                            title="'. $data_arr['cell_count'] .' '. $this->getXCStr($state_type) .' cells">
                          <span>'. $data_arr['percentage'] .'%</span>
                      </div>';

                  $z_index--;
              }
          }

          // pick up total count for this step
          $total_count_arr[$step_nr] = $state_arr['total_count'];
      }

      // create data rows
      $state_data_td_arr = array();
      foreach (range($this->getNumberOfXc(), 0) as $state) {

          $state_data_tds = '';
          foreach (range(0, $this->experiment->getNumberOfDays()) as $step_nr) {

              // check if this step is logged at all
              if (!isset($exp_data_arr[$step_nr])) continue;

              // check if this step is logged
              $cell_count = 0;
              if (isset($exp_data_arr[$step_nr][$state])) {

                  // add all cells per state (group per sub state)
                  foreach ($exp_data_arr[$step_nr][$state] as $state_type=>$state_type_arr) {
                      $cell_count += $state_type_arr['cell_count'];
                  }
              }

              $state_data_tds .= '<td>'. $cell_count .'</td>';
          }

          $state_data_td_arr[] = $state_data_tds;
      }

      if (!empty($state_graph_arr)) {
          $html .= '
              <table class="result">
                  <tr class="steps">
                      <th>'. implode('</th><th>', array_keys($state_graph_arr)) .'</th>
                  </tr>
                  <tr class="bars">
                      <td><div class="step-wrapper">'. implode('</div></td><td><div class="step-wrapper">', $state_graph_arr) .'</div></td>
                  </tr>
                  <tr class="data">'. implode("</tr>\n\t\t\t\t\t<tr class=\"data\">", $state_data_td_arr) .'</tr>
                  <tr class="total-counts">
                      <td>'. implode('</td><td>', $total_count_arr) .'</td>
                  </tr>
              </table>';
      }

      return $html;
  }

  /**
   * Start the experiment
   *    * @access private
   * @return void
   */
  private function startExperiment()
  {
      // guard: check for experiment
      if (!$this->experiment) return;

      // initialize cells
      $this->initCells();

      //initialize experiment step counter
      self::$step_nr = 0;

      $this->logCurrentState($clear_state=TRUE);

      // loop through the experiment steps
      foreach (range(1, $this->experiment->getNumberOfDays()) as $step_nr) {

          // set global step number
          self::$step_nr = $step_nr;

          // process the inactivation
          if (self::$mode == 1) {
              $this->processInactivationModeI();
          } elseif(self::$mode == 2) {
              $this->processInactivationModeII();
          }

          // duplicate the cells. Rate per cell-state,           // deelt als leeft (stop flag is 0)
          foreach ($this->duplication_arr as $state=>$duplication) {
              if ($duplication_rate = $duplication->getRate()) {
                  if (($step_nr % $duplication_rate) == 0) {
                      //echo 'duplicating state '. $state .' in step '. $step_nr .'<br />';
                      $this->duplicateCells($state);
                  }
              }
          }

          // log the cell's state
          $this->logCurrentState();
      }
  }

  /**
   * Process the inactivation
   *    * @access private
   * @return void
   */
  private function processInactivationModeI()
  {
      // 1 - EXPERIMENT STEP PER X
      $xci_sql_arr = array();
      foreach (range(1, $this->getNumberOfXc()) as $i) {

          if ($inactivation = $this->inactivation_arr[$i]) {
              $inactivation_rate = $inactivation->{'rate_step'.self::$step_nr};
              $xci_sql_arr[] = 'x.xc'. $i .' = x.xc'. $i .'*(100*RAND()>'. round($inactivation_rate) .')';
          }
      }

      // guard
      if (empty($xci_sql_arr)) return;

      $sql = "
          UPDATE xci_set x
            SET
              ". implode(', ', $xci_sql_arr) ."
          WHERE x.stop_flag = 0;
      ";
      // echo  $sql;
      $this->logSQL($sql);
      Record::getRecordsByQuery($sql);

      $this->markDeadCells();
  }

  /**
   * Process the inactivation with a single curve and thresholds
   *    * @access private
   * @return void
   */
  private function processInactivationModeII()
  {
      // 1 - EXPERIMENT STEP PER STATE PER X
      // for every state process inactivation
      // the duplication_arr is ordered by state ASC and contains (per state) the downscale factor for the inactivation curve
      // the states are processes ascending such that the sequential steps don't overlap (state 2 never becomes 3, only 2 or 1 or 0)
      foreach ($this->duplication_arr as $state => $duplication) {

          $xci_sql_arr = array();
          foreach ($this->inactivation_arr as $xci=>$inactivation) {

              $scale     = (float) $duplication->getScaleFactor();
              $rate      = (int) $this->inactivation_curve->{'rate_step'.self::$step_nr};
              $threshold = (int) $inactivation->getThreshold();

              $inactivation_rate = round(max(($scale * $rate) - $threshold, 0));

              $xci_sql_arr[] = 'x.xc'. $xci .' = x.xc'. $xci .'*(100*RAND()>'. $inactivation_rate .')';

              /*/
              $log = '
                  step: '. self::$step_nr .', state '. $state .', Xi: '. $xci .'
                  (scale: '. $scale .' * rate: '. $rate .') - threshold: '. $threshold .' = gives irate: '. $inactivation_rate .'
                  -------------------------------------------------------------
              ';
              echo $log; /* */
              /*/
              $log = '
              i'. self::$step_nr .'Xa'. $state .'r'. $inactivation_rate .'<br />';
              echo $log; /* */
          }

          // loop guard
          if (empty($xci_sql_arr)) return;

          $sql = "
              UPDATE xci_set x
                SET
                  ". implode(', ', $xci_sql_arr) ."
              WHERE                   (". implode(' + ', $this->getXCArr()) .") = ". $state ." AND
                  x.stop_flag = 0;
          ";
          //echo ($state < 3) ? $sql : '';

          $this->logSQL($sql);
          Record::getRecordsByQuery($sql);
      }

      $this->markDeadCells();
  }

  /**
   * Take 'inactivated' Cells Out Of Experiment
   *    * @access private
   * @return void
   */
  private function markDeadCells()
  {
      $sql = "
          UPDATE xci_set x
            SET x.stop_flag = 1
          WHERE (". implode(' + ', $this->getXCArr()) .") <= ". ceil($this->getNumberOfXc()/2).";
      ";
      // echo  $sql;
      $this->logSQL($sql);
      Record::getRecordsByQuery($sql);
  }

  /**
   * Duplicate the living cells
   *
   * @param int state
   * @access private
   * @return void
   */
  private function duplicateCells($state)
  {
      // init cell state
      $xc_arr = $this->getXCArr();

      // 3 - DUPLICATE LIVING CELLS
      $sql = "
          INSERT INTO xci_set (stop_flag, ". implode(', ', $xc_arr) .")
          SELECT
            stop_flag, ". implode(', ', $xc_arr) ."
          FROM
            xci_set x
          WHERE
            ". implode(' + ', $xc_arr) ." = ". $state ."
      ";

      // echo  $sql;
      $this->logSQL($sql);
      Record::getRecordsByQuery($sql);
  }

  /**
   * Log the current state
   *
   * @param bool Whether or not to clear the existing log entries of this experiment
   * @access private
   * @return void
   */
  private function logCurrentState($clear_state=FALSE)
  {
      // delete old entries
      if ($clear_state) {
          $sql = "
              DELETE FROM                   xci_set_log". self::$mode ."
              WHERE
                  experiment_id = ". $this->experiment->getId() ."
          ";
          // echo $sql;
          $this->logSQL($sql);
          Record::getRecordsByQuery($sql);
      }

      $state_type_select_arr = array();
      $i = 1;
      foreach($this->getXCArr() as $xci) {
          // create array like array('1*xc1', '2*xc2', '4*xc3'), like CHMOD 755, 7 stands for 111 binary, so all states active
          $state_type_select[] = $i.'*'.$xci;
          $i *= 2;
      }

      $sql = "
          INSERT INTO xci_set_log". self::$mode ." (`experiment_id`, `experiment_step`, `cell_state`, `state_type`, `state_count`)
          SELECT
            ". $this->experiment->getId() .",
            ". self::$step_nr .",
            ". implode(' + ', $this->getXCArr()) ." as cell_state,
            ". implode(' + ', $state_type_select) ." as state_type,
            count(". implode(' + ', $this->getXCArr()) .") as state_count
          FROM
            xci_set x
          GROUP BY ". implode(', ', $this->getXCArr()) .", cell_state
          ORDER BY cell_state DESC, state_type DESC
      ";
      // echo  $sql;
      $this->logSQL($sql);
      Record::getRecordsByQuery($sql);

      /*/ Without sub states
          SELECT
            101,
            3,
            xc1 + xc2 as cell_state,
            count(xc1 + xc2) as state_count
          FROM
            xci_set x
          GROUP BY cell_state
          ORDER BY cell_state DESC;
       /* */

       /*/ With sub states marked binary
          SELECT
            101,
            3,
            xc1 + xc2 + xc3 as cell_state,
            1*xc1 + 2*xc2 + 4*xc3 state_type,
            count(xc1 + xc2 + xc3) as state_count
          FROM
            xci_set x
          GROUP BY xc1, xc2, xc3, cell_state
          ORDER BY cell_state DESC, state_type ASC;
      /* */
  }

  /**
   * Initiate the cell set
   *
   * @access private
   * @return void
   */
  private function initCells()
  {
      // guard: number of x chromo's must be at least 2
      if ($this->getNumberOfXc() < 2) return;

      // clear current state
      $sql = "TRUNCATE xci_set";
      $this->logSQL($sql, 0);
      Record::getRecordsByQuery($sql);

      $sql = "ALTER TABLE `xci`.`xci_set` AUTO_INCREMENT = 1";
      $this->logSQL($sql, 0);
      Record::getRecordsByQuery($sql);

      $sql = "
          INSERT INTO
              xci_set (stop_flag,". implode(',', $this->getXCArr()) .")
          SELECT
              stop_flag,
              ". implode(',', $this->getXCArr()) ."
          FROM
              xci_set_start
          LIMIT ". $this->experiment->getNumberOfCells() ."
      ";
      //echo $sql;
      $this->logSQL($sql, 0);
      Record::getRecordsByQuery($sql);
  }

  /**
   * Log an SQL query
   *
   * @param string SQL
   * @access private
   * @return void
   */
  private function logSQL($sql)
  {
      return;

      $log = Record::create('xc_sql_log');
      $log->setExperimentId($this->experiment->getId());
      $log->setExperimentStep(self::$step_nr);
      $log->setSQL($sql);
      $log->save();
  }

  /**
   * Get an array with the state names
   *
   * @access private
   * @return array
   */
  private function getXCArr()
  {
      $xc_arr = array();
      foreach (range(1, $this->getNumberOfXc()) as $i) {
          $xc_arr[$i] = 'xc'.$i;
      }

      return $xc_arr;
  }

  /**
   * Get an array with the state names
   *
   * @param int state type
   * @access private
   * @return array
   */
  private function getXCStr($state_type)
  {
      $xc_str = '';
      foreach (range(0, ($this->getNumberOfXc()-1)) as $i) {
          //echo $i .': '. pow(2,$i) .' & '.  $state_type .' : '. (pow(2,$i) & $state_type) .'<br />';
          if (pow(2,$i) & $state_type) {
              $xc_str .= 'Xa';
          } else {
              $xc_str .= 'Xi';
          }
      }

      return $xc_str;
  }

  /**
   * Get the number of X Chromosomes used in the experiment, equals the number of linked inactivation arrays
   *
   * @access private
   * @return int Number of experiments
   */
  private function getNumberOfXc()
  {
      return count($this->inactivation_arr);
  }

  /**
   * Fetch the experiment data in a nice array
   *
   * @access private
   * @return string HTML
   */
  private function getExperimentDataArr($mode)
  {
      $exp_data_arr = array();

      $sql = "
          SELECT
            [l1.id](http://l1.id/),
            l1.experiment_step,
            l1.cell_state,
            l1.state_type,
            l1.state_count,
            SUM(l2.state_count) as total_count
          FROM
            xci_set_log". $mode ." l1
          JOIN xci_set_log". $mode ." l2 ON
            l1.experiment_step = l2.experiment_step AND
            l2.experiment_id = ". $this->experiment->getId() ."
          WHERE
            l1.experiment_id = ". $this->experiment->getId() ."
          GROUP BY l1.state_type, l1.experiment_step
          ORDER BY l1.cell_state DESC, l1.state_type DESC
      ";
      //echo  $sql;
      $state_log_arr = Record::getRecordsByQuery($sql);

      // transform result set into a nice accessable format: array[step_nr][state]
      $unit_height = 250;
      foreach($state_log_arr as $state_log) {

          $step_nr    = $state_log->getExperimentStep();
          $state      = $state_log->getCellState();
          $cell_cnt   = $state_log->getStateCount();
          $state_type = $state_log->getStateType();
          $total_cnt  = $state_log->getTotalCount();

          $state_type_arr['height']     = number_format($unit_height*$cell_cnt/$total_cnt, 1);
          $state_type_arr['percentage'] = number_format(100*$cell_cnt/$total_cnt, 1);
          $state_type_arr['cell_count'] = $cell_cnt;

          $exp_data_arr[$step_nr][$state][$state_type]  = $state_type_arr;
          $exp_data_arr[$step_nr]['total_count']        = $total_cnt;
      }

      return $exp_data_arr;
  }

  /**
   * Content getter
   *    * @access public
   * @return string HTML
   */
  public function getNavigationMain()
  {
      $exp_data_arr = Record::getRecords('xci_experiments', array(), 'id ASC');

      $list_items = '';
      foreach ($exp_data_arr as $experiment) {

          $class_str = '';
          $classes_arr = array();
          if ($this->experiment && ($this->experiment->getId() == $experiment->getId())) {
              $classes_arr[] = 'sel';
          }
          $class_str = !empty($classes_arr) ?  ' class="'. implode(' ', $classes_arr) .'"' : '';

          $list_items .= '
              <li'. $class_str .'>
                  <a href="'. $this->section->uri .'/'. $experiment->getId() .'">'. $experiment->getName('text') .'</a>
              </li>';
      }

      $html = '
          <ul id="nav-main">
              '. $list_items .'
          </ul>';

      return $html;
  }
}

?>

--
