# Supplementary figures and images for: The Probability to Initiate X Chromosome Inactivation Is Determined by the X to Autosomal Ratio and X Chromosome Specific Allelic Properties
Source: PLoS One. 2009 May 19;4(5):e5616. doi: 10.1371/journal.pone.0005616 (PMC2680018; doi:10.1371/journal.pone.0005616)

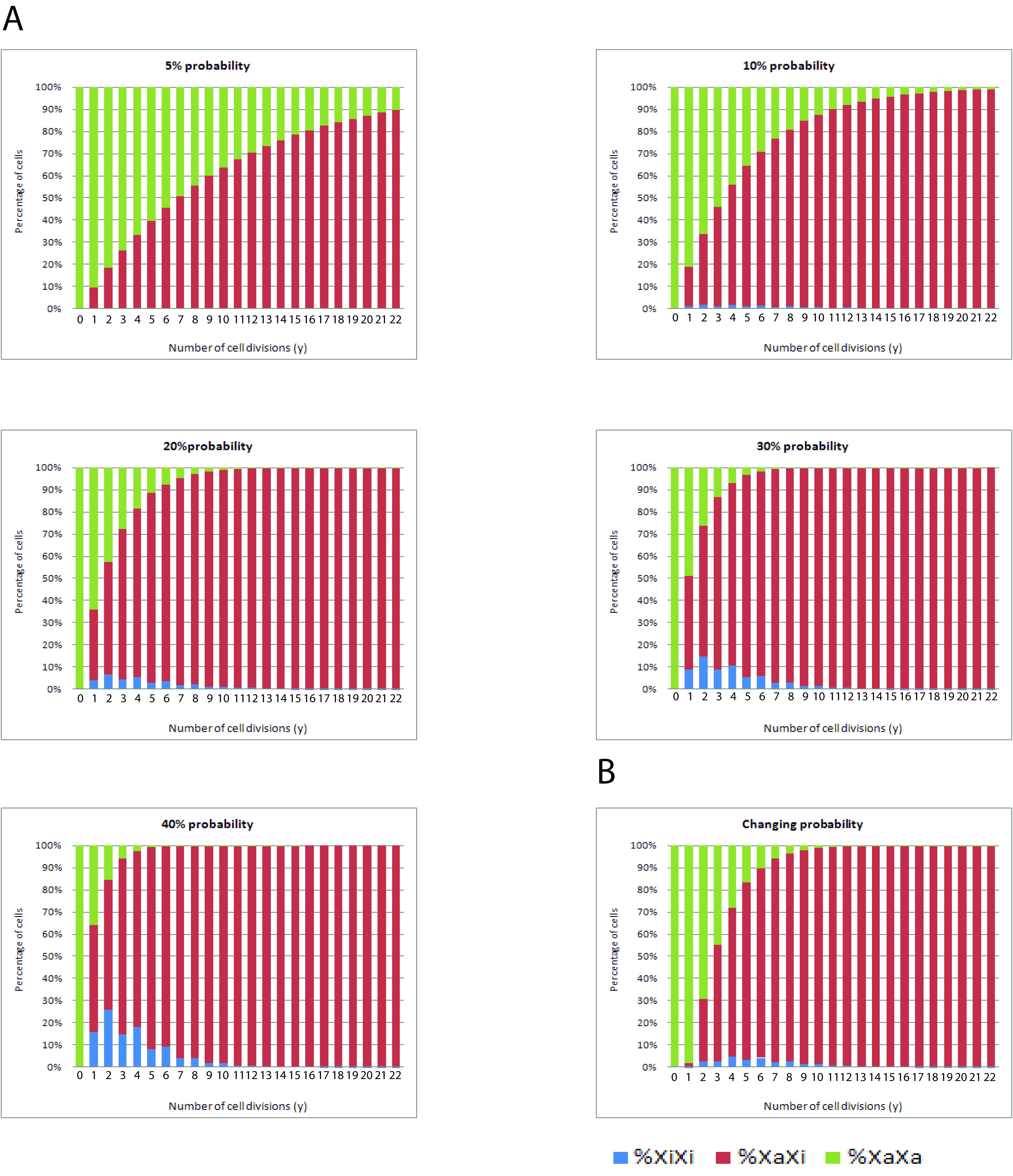

Supplement: Figure S1 — Mathematical computation of cell populations A) The panels show the mathematical computation the XaXa, XaXi and XiXi populations with a 5%, 10%, 20%, 30%, and 40% fixed probability per X chromosome. The different bar-graphs show the relative distribution of the three different cell types (XaXa = green, XiXa = red, XiXi = blue), in a 22 day differentiation experiment. B) This panel shows the mathematical computation the XaXa, XaXi and XiXi populations with a changing probability for m = 1 presented in figure 4B. (3.11 MB TIF) [file pone.0005616.s002.tif]

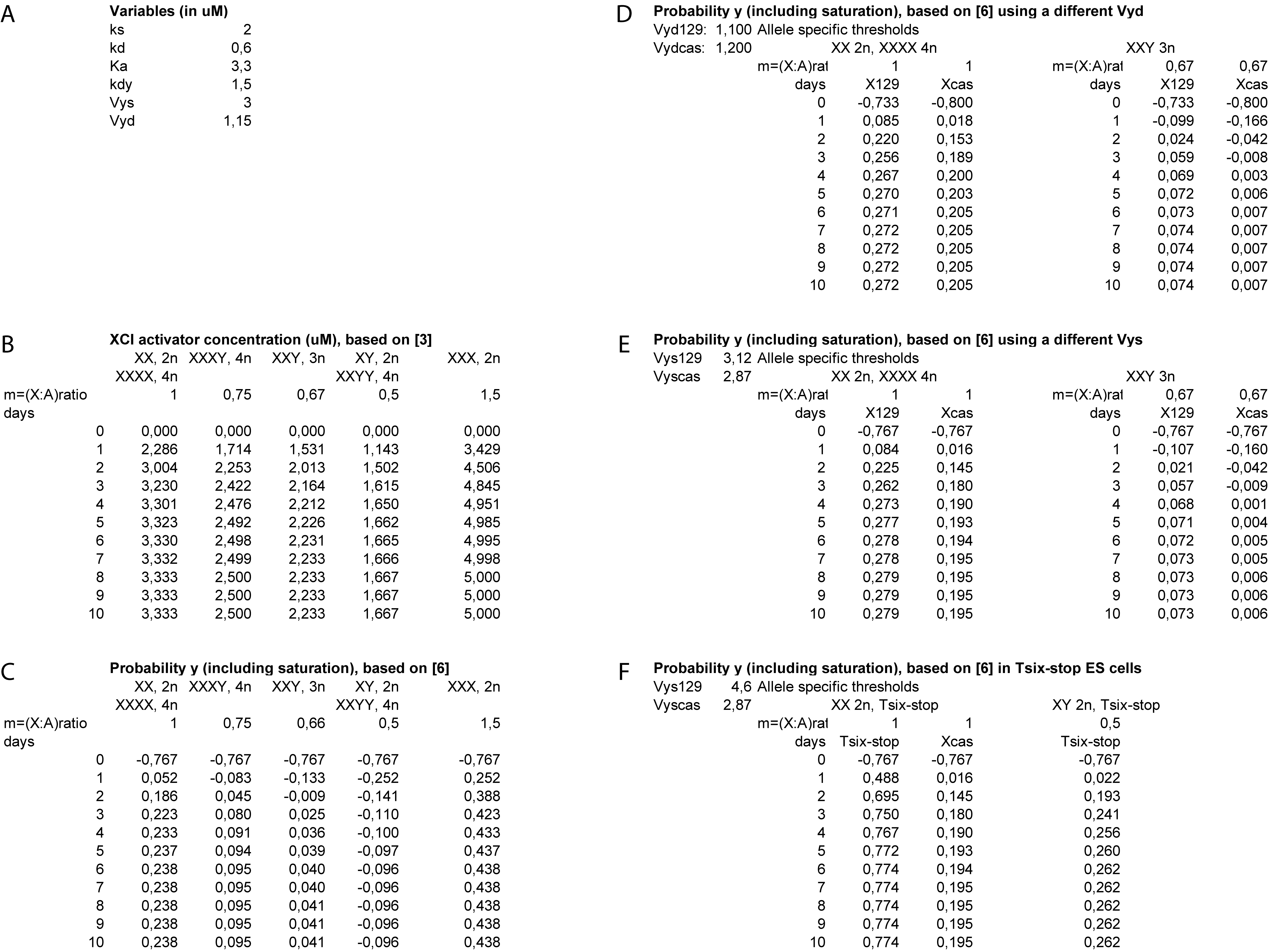

Supplement: Figure S4 — Calculation of the probability y A, B) This figure shows (B) the XCI-activator concentration in a nucleus with a different X∶A ratio (m), based on values for the different variables given in (A). (C) The probability y was determined for cells with a different number of sex chromosomes and/or ploidy. D, E, F) Show the allele specific probability y in time with different Vyd or Vys values in wild type (D, E) and Tsix-stop cells (F), used in our simulation experiments. (1.84 MB TIF) [file pone.0005616.s005.tif]

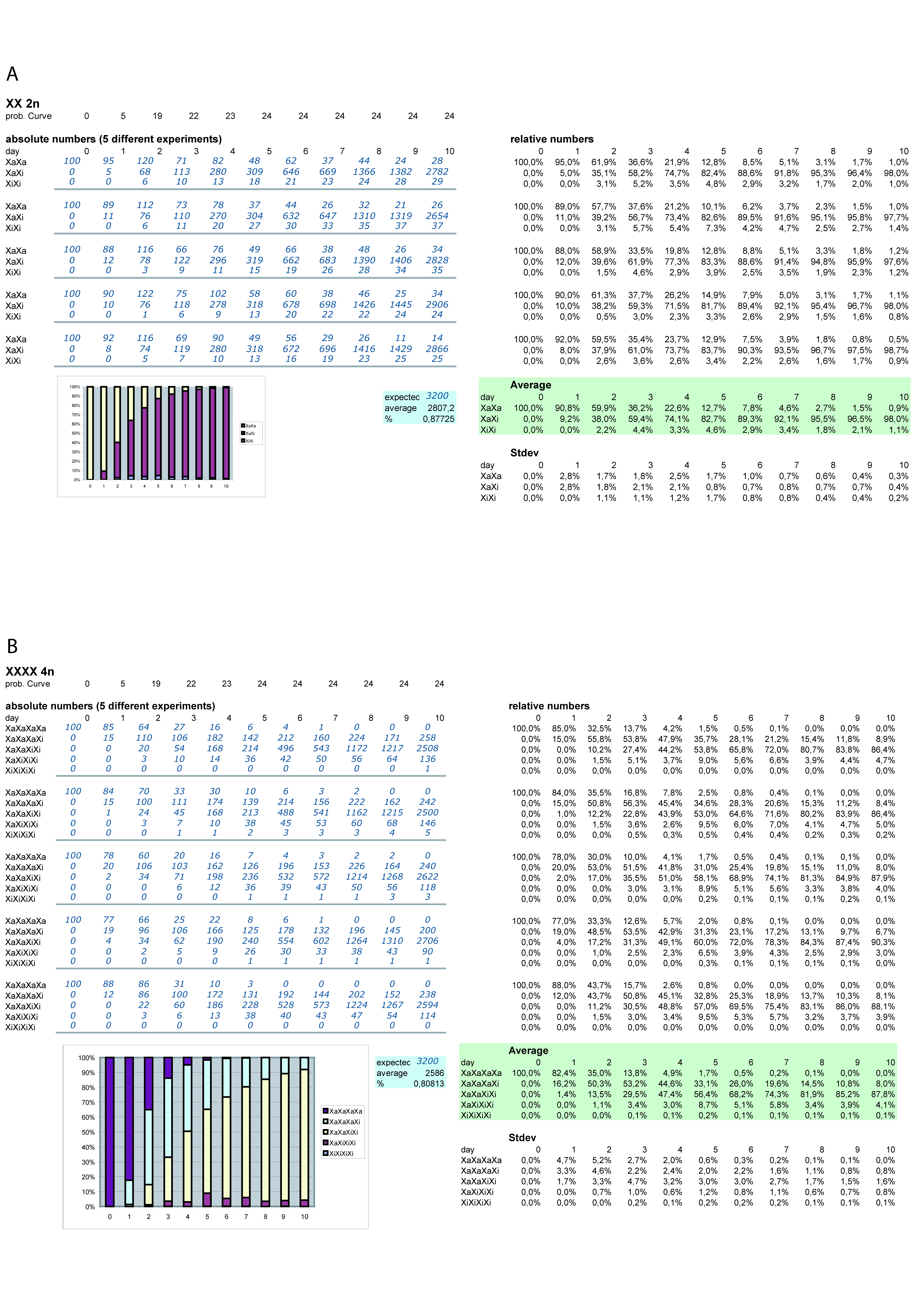

Supplement: Figure S5 — Stochastic simulation of XCI in diploid XX and tetraploid XXXX cells A, B) Results of the stochastic simulations using the probability curves shown in Figure 4B for diploid XX (A) and tetraploid XXXX cells (B). The average of five independent runs is highlighted in green. The expected number of XiXa and XiXiXaXa cells and the experimentally obtained number of XiXa and XiXiXaXa cells from the diploid and tetraploid cells respectively are highlighted in blue. (3.07 MB TIF) [file pone.0005616.s006.tif]

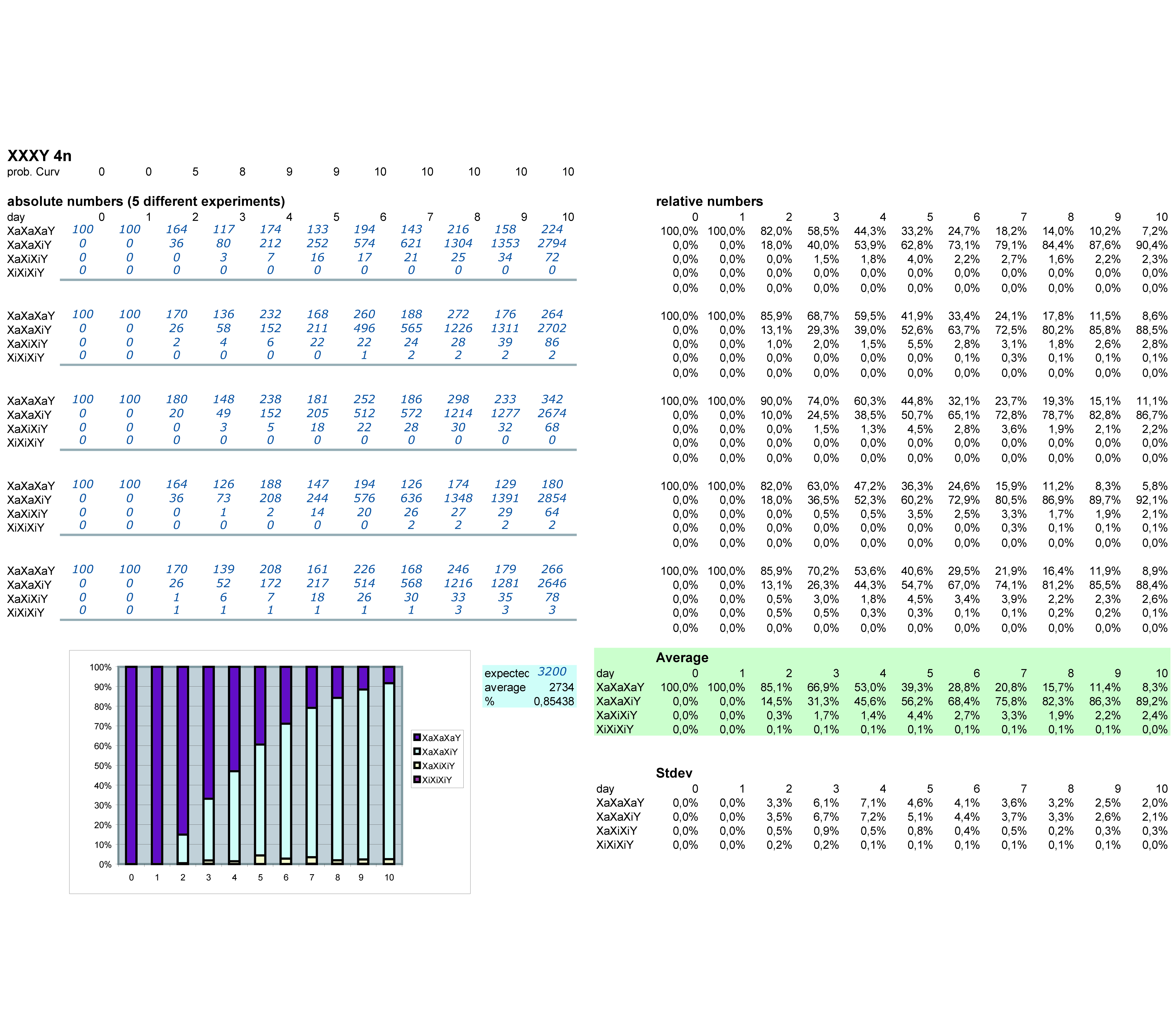

Supplement: Figure S6 — Stochastic simulation of XCI in tetraploid XXXY cells Results of the stochastic simulations using the probability curves shown in Figure 4B for tetraploid XXXY cells. The average of five independent runs is highlighted in green. The expected and obtained number of tetraploid XiXaXaY cells are highlighted in blue. (1.77 MB TIF) [file pone.0005616.s007.tif]

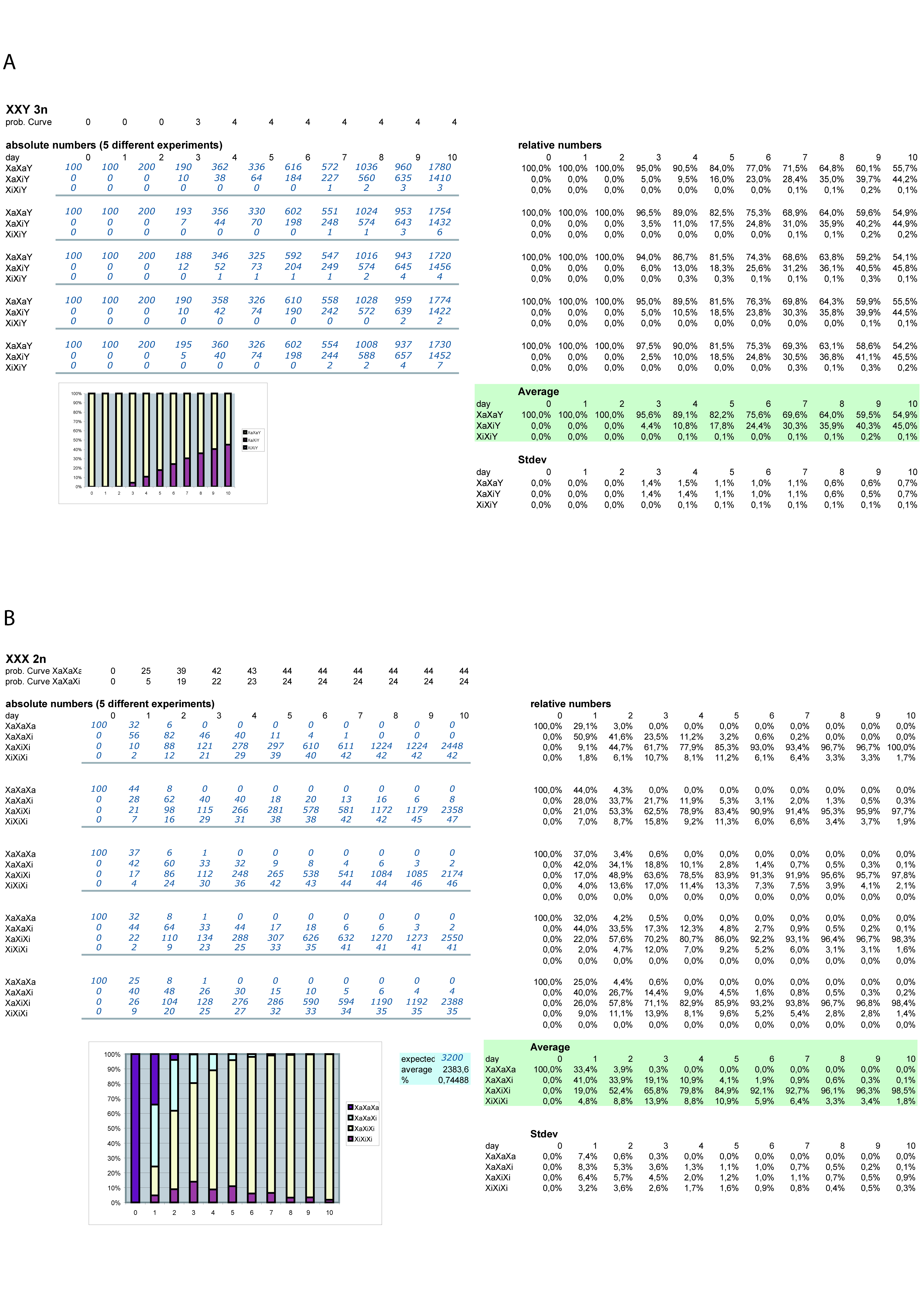

Supplement: Figure S7 — Stochastic simulation of XCI in triploid XXY and diploid XXX cells Results of the stochastic simulations using different probability curves presented in Figure 4D for triploid XXY cells (A) and diploid XXX cells (B). The average of five independent runs is highlighted in green. Except for the triploid XXY cells, the expected and obtained number of viable cells is highlighted in blue. (2.70 MB TIF) [file pone.0005616.s008.tif]

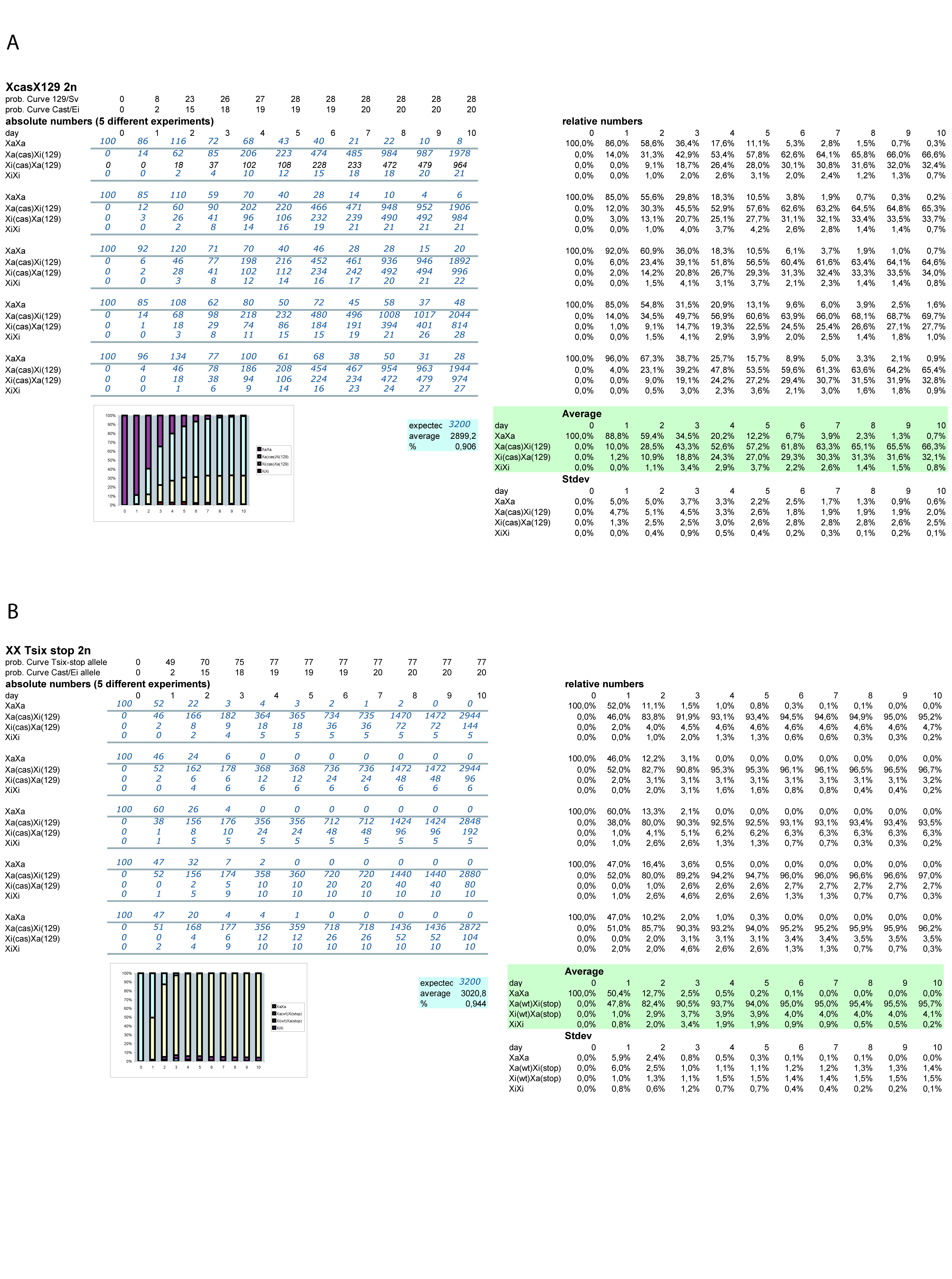

Supplement: Figure S8 — Stochastic simulation of XCI in diploid cells with allele specific probabilities A, B) Results of stochastic simulations using the X∶A ratio of 1, and allele specific probabilities indicated in Figure 5A (A) and 5C (B). (A) shows the simulation of F1 female Cast/Ei 129/Sv cells, (B) heterozygous female Tsix-stop cells. The average of five independent runs is highlighted in green. The expected and obtained number of viable cells are highlighted in blue. (2.68 MB TIF) [file pone.0005616.s009.tif]

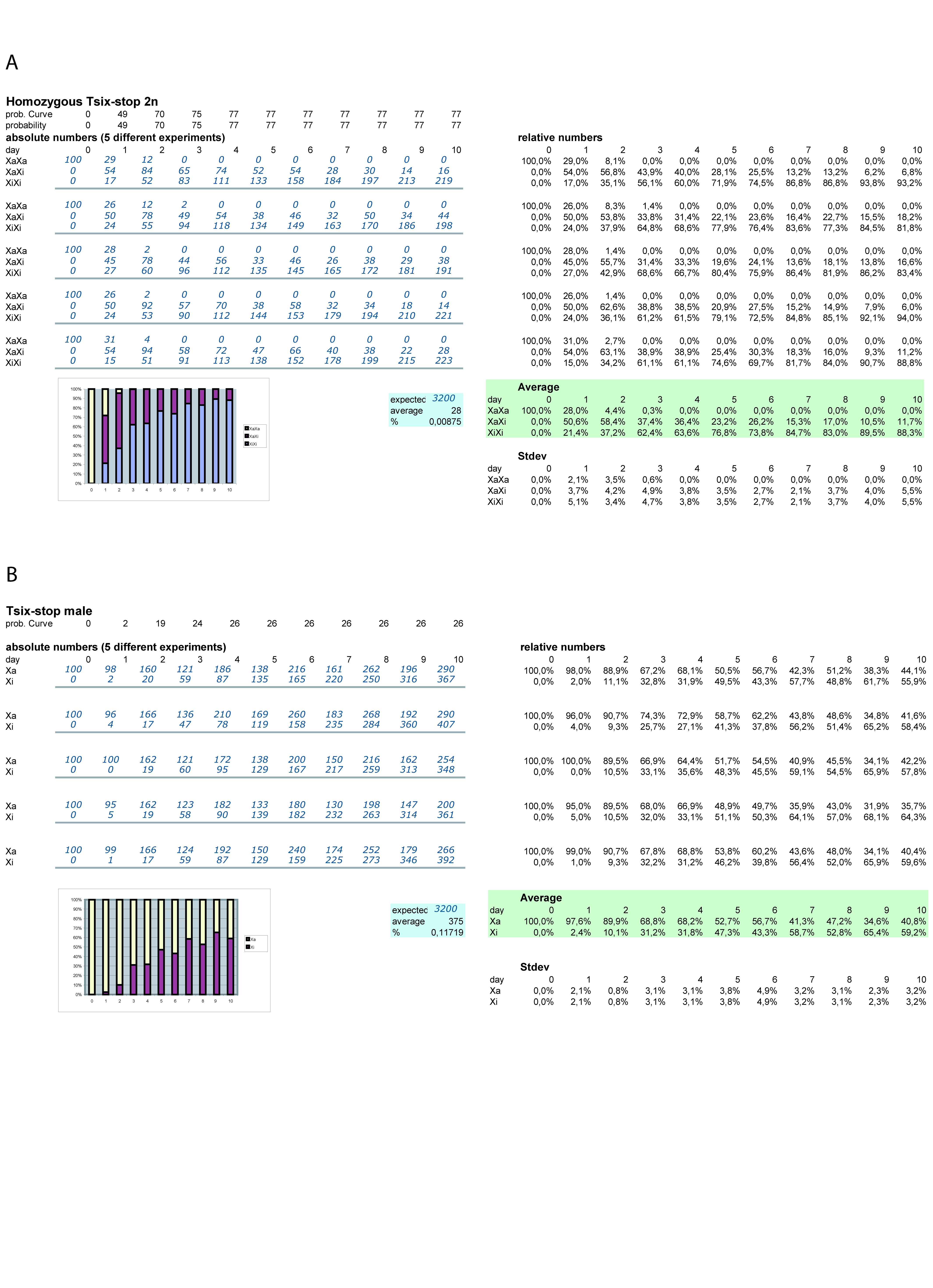

Supplement: Figure S9 — Stochastic simulation of XCI in female and male cells with a Tsix-stop allele A, B) Results of stochastic simulations using the X∶A ratio of 1, and allele specific probabilities indicated in Figure 5E. Simulation experiments with homozygous female (A) and hemizygous male (B) Tsix-stop alleles. The average of five independent runs is highlighted in green. The expected and obtained number of viable cells are highlighted in blue. (2.46 MB TIF) [file pone.0005616.s010.tif]
